# Sensing the Spin State of Room-Temperature Switchable Cyanometallate Frameworks with Nitrogen-Vacancy Centers in Nanodiamonds

**DOI:** 10.1021/acsnano.3c11820

**Published:** 2024-02-21

**Authors:** Bradley T. Flinn, Graham A. Rance, William J. Cull, Ian Cardillo-Zallo, Jem Pitcairn, Matthew J. Cliffe, Michael W. Fay, Ashley J. Tyler, Benjamin L. Weare, Craig T. Stoppiello, E. Stephen Davies, Melissa L. Mather, Andrei N. Khlobystov

**Affiliations:** † School of Chemistry, University of Nottingham, Nottingham, NG7 2RD, United Kingdom; ‡ Nanoscale and Microscale Research Centre, University of Nottingham, Nottingham, NG7 2RD, United Kingdom; § School of Chemistry, University of Birmingham, Birmingham, B15 2TT, United Kingdom; ∥ Optics and Photonics Group, Faculty of Engineering, University of Nottingham, Nottingham, NG7 2RD, United Kingdom; ⊥ Centre for Microscopy and Microanalysis, University of Queensland, St. Lucia, 4072, Australia

**Keywords:** Nitrogen-vacancy sensing, photomagnetism, spin-crossover, nanodiamond, metal−organic framework, transmission electron microscopy

## Abstract

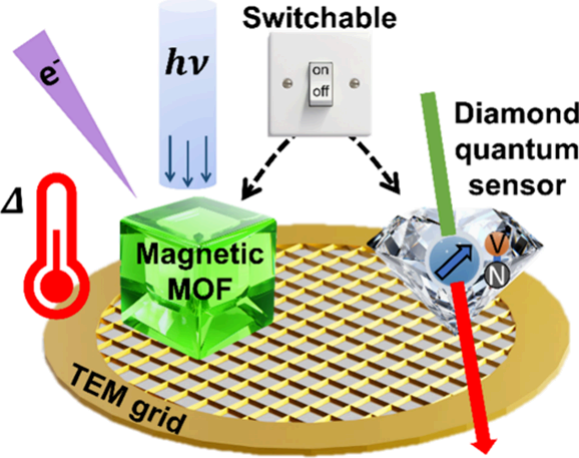

Room-temperature magnetically switchable materials play a vital role in current and upcoming quantum technologies, such as spintronics, molecular switches, and data storage devices. The increasing miniaturization of device architectures produces a need to develop analytical tools capable of precisely probing spin information at the single-particle level. In this work, we demonstrate a methodology using negatively charged nitrogen vacancies (NV^–^) in fluorescent nanodiamond (FND) particles to probe the magnetic switching of a spin crossover (SCO) metal–organic framework (MOF), [Fe(1,6-naphthyridine)_2_(Ag(CN)_2_)_2_] material (**1**), and a single-molecule photomagnet [X(18-crown-6)(H_2_O)_3_]Fe(CN)_6_·2H_2_O, where X = Eu and Dy (materials **2a** and **2b**, respectively), in response to heat, light, and electron beam exposure. We employ correlative light–electron microscopy using transmission electron microscopy (TEM) finder grids to accurately image and sense spin–spin interacting particles down to the single-particle level. We used surface-sensitive optically detected magnetic resonance (ODMR) and magnetic modulation (MM) of FND photoluminescence (PL) to sense spins to a distance of ca. 10–30 nm. We show that ODMR and MM sensing was not sensitive to the temperature-induced SCO of Fe^II^ in **1** as formation of paramagnetic Fe^III^ through surface oxidation (detected by X-ray photoelectron spectroscopy) on heating obscured the signal of bulk SCO switching. We found that proximal FNDs could effectively sense the chemical transformations induced by the 200 keV electron beam in **1**, namely, Ag^I^ → Ag^0^ and Fe^II^ → Fe^III^. However, transformations induced by the electron beam are irreversible as they substantially disrupt the structure of MOF particles. Finally, we demonstrate NV^–^ sensing of reversible photomagnetic switching, Fe^III^ + (18-crown-6) ⇆ Fe^II^ + (18-crown-6)^+ •^, triggered in **2a** and **2b** by 405 nm light. The photoredox process of **2a** and **2b** proved to be the best candidate for room-temperature single-particle magnetic switching utilizing FNDs as a sensor, which could have applications into next-generation quantum technologies.

Switchable magnetic materials, such as photomagnetic and spin crossover (SCO) molecules, have been considered as promising materials for application in memory storage, spintronics, and molecular switching nanodevices.^1,2^ The ability to switch materials at ambient conditions with an external stimulus (light, heat, etc.), for example, in SCO complexes, is especially desirable for most device-type applications. Cyanometallates show promise due to their tunable crystallite size, morphology, and controllable magnetic interactions.^3^ The most widely studied SCO chemistry involves octahedral Fe^II^ centers which have two switchable spin-states, low-spin (LS, *S* = 0, no unpaired d-orbital electrons) and high-spin (HS, *S* = 2, 4 unpaired d-orbital electrons). Hiiuk et al. synthesized a Hoffmann-like polymeric Fe, Ag cyanoheterometallic compound with hysteretic SCO centered at room temperature (297 K).^4^ Also, Cai et al. have recently synthesized a variety of photomagnetic 3d–4f hexacyanoferrate single-molecule magnets which, upon light illumination at room temperature, exhibit large changes in their magnetic properties, including photoinduced magnetic phase transitions and large (de)magnetization effects.^5,6^ A less commonly employed stimulus that can change the chemical and magnetic state of materials is an electron beam (e^–^ beam).^7^ The e^–^ beam is widely used in electron microscopy to investigate the state of a material, but it can be an invasive probe that results in irreversible physicochemical alteration of materials (especially for those known to be sensitive to the e^–^ beam).^8,9^ Electron microscopy imaging and integrated elemental spectroscopy are often combined to characterize such processes. Monitoring magnetic transformations within samples upon e^–^ beam irradiation at the nanoscale is challenging, as electrons interact much more weakly with the magnetic moment of electrons than their charge: with advanced techniques such as Lorentz microscopy required to image magnetic features, including ferromagnet domain structures.^10^

Most magnetic instrumentation with sufficient sensitivity to investigate magnetic switchability often feature macroscopically sized sensing elements, such as pick-up coils (in superconducting quantum interference devices, SQUIDs) and Hall bars (Hall probes), and as such, appreciable sample volumes are often required. In addition, many of these instruments have stringent requirements on operating conditions, such as the need for superconducting temperatures with SQUID on a tip methodologies.^11^ For most magnetic quantum technological applications, it is desirable to miniaturize spin-detection probe and target spin volumes, operating under ambient conditions. Negatively charged nitrogen-vacancy (NV^–^) defects imbedded into diamond matrices have optically addressable spins, at room temperature, which are highly sensitive to a diverse range of changes in environmental conditions, including high-frequency magnetic fields associated with the fluctuations of proximal paramagnetic spins.^12,13^ The NV center is a naturally occurring impurity comprising a substitutional nitrogen atom next to a vacant site in the diamond lattice, which, in the negative charge state, forms a spin triplet, *m*_*s*_ = −1, 0, and +1 (Figures 1B and 1C).^14,15^ The NV^–^ center has attracted attention as a potential fluorescent probe for use in quantum technological applications, due to its high quantum yield and robust photoluminescence (PL).^16,17^ It also provides a detection platform for switchable spin states of materials down to the nanoscale. A recent study by Lamichhane et al. employs a NV^–^ implanted macro-diamond substrate to study the magnetic properties of SCO Fe^II^-triazole nanoparticle clusters and individual nanorods by correlating with scanning electron microscopy (SEM).^18^ Fluorescent nanodiamonds (FNDs) are advantageous as they provide an alternative platform for NV^–^ sensing, allowing the sensor to be incorporated into a wide range of systems, such as in cell biology and quantum technology devices, for a diverse range of applications.^19−21^ Recently, Flinn et al. have demonstrated FNDs as local probes for the detection of spin noise within paramagnetic nanoparticles, establishing the strength of NV^–^ sensing response as a function of distance and local concentration.^22^ The methodology reported performed ODMR in a regime where the optically induced NV polarization rate is influenced by intrinsic and external spin–lattice relaxation processes, leading to changes in ODMR contrast. A protocol using a strong external magnetic field, causing significant spin state mixing in both the ground and excited NV levels was also reported. This magnetic field was sufficiently strong to define the quantization axis and an increase in nonradiative intersystem crossing and reduced photoluminescence intensity. Diminished spin polarization in the presence of paramagnetic spin noise was demonstrated, resulting in reduced PL contrast with magnet amplitude modulation.^22^ However, the challenge of sensing magnetic state switching at the single-particle level using FNDs remains unaddressed.

**Figure 1:**
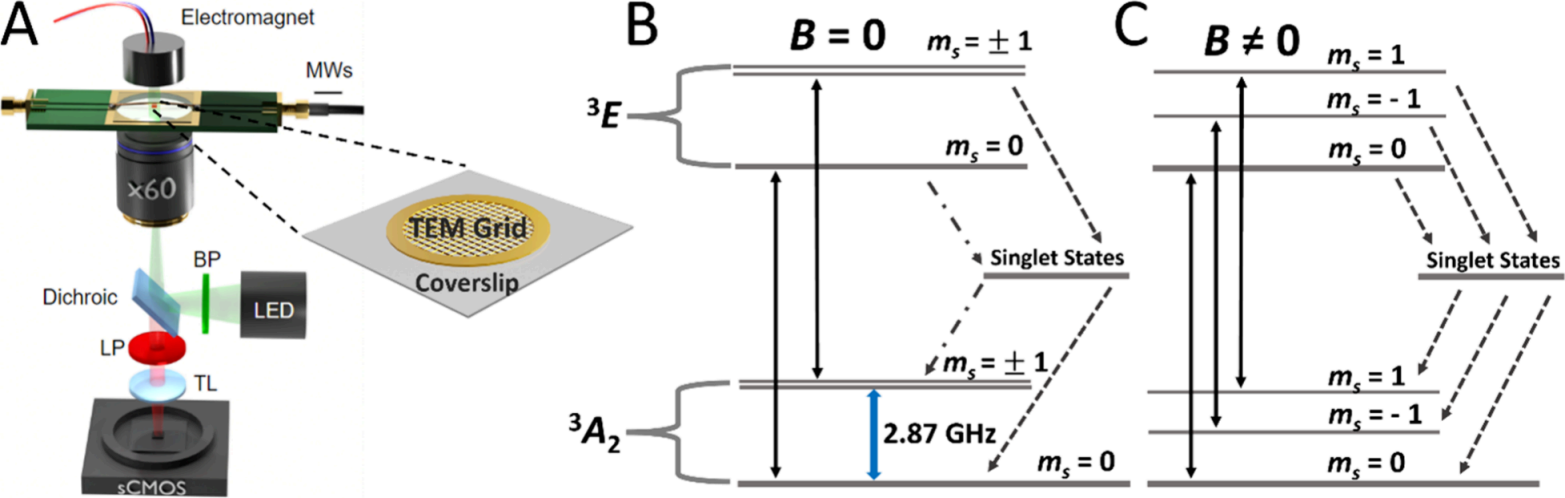
(A) Schematic experimental inverted fluorescence microscope setup. FNDs are adsorbed directly onto TEM grids or glass coverslips. A custom-made PCB with a wire antenna was used for microwave delivery and an off NV axis electromagnet placed directly above the experimental setup was used to modulate magnetic field. Light illumination and detection pathways are also shown. Schematic Jablonski diagrams show the excitation and decay pathways of the NV^–^ center (B) without and (C) with the application of the off-axis magnetic field. Transitions between ground and excited triplet states (solid arrows), as well as microwave stimulated relaxations, are shown with nonradiative singlet state pathways, weak (**− • –**) and strong (**−**) transitions, highlighted.

Herein, we demonstrate the use of NV^–^ centers in FNDs to probe three types of room-temperature transformations in magnetic materials, at the single-particle level, initiated by heat, light, and an e^–^ beam. The readout, using FNDs, of switching induced by heat and e^–^ beam irradiation was investigated and demonstrated, respectively, in an Fe^II^ SCO material ([Fe(1,6-N)_2_(Ag(CN)_2_)_2_] – 1,6-N = 1,6-naphthyridine, material **1**, and demonstrated (reversibly) by light illumination in 3d–4f lanthanide crown ether hexacyanoferrates ([X(18C6)(H_2_O)_3_]Fe(CN)_6_·2H_2_O – 18C6 = 18-crown-6, where X = Eu or Dy (materials **2a** and **2b**, respectively) (see Table 1). By employing Raman spectroscopy, SQUID magnetometry, and X-ray photoelectron spectroscopy (XPS) combined with a recently demonstrated correlative light-electron NV^–^ sensing method for detecting local external fluctuating magnetic spin noise,^22^ we investigate the relationship between surface and bulk chemical and magnetic transformations down to the single-particle level. This work could be employed for the future characterization of switchable magnetic materials and demonstrates a workflow to unlock vital information for application into quantum technology.

**Table 1:**
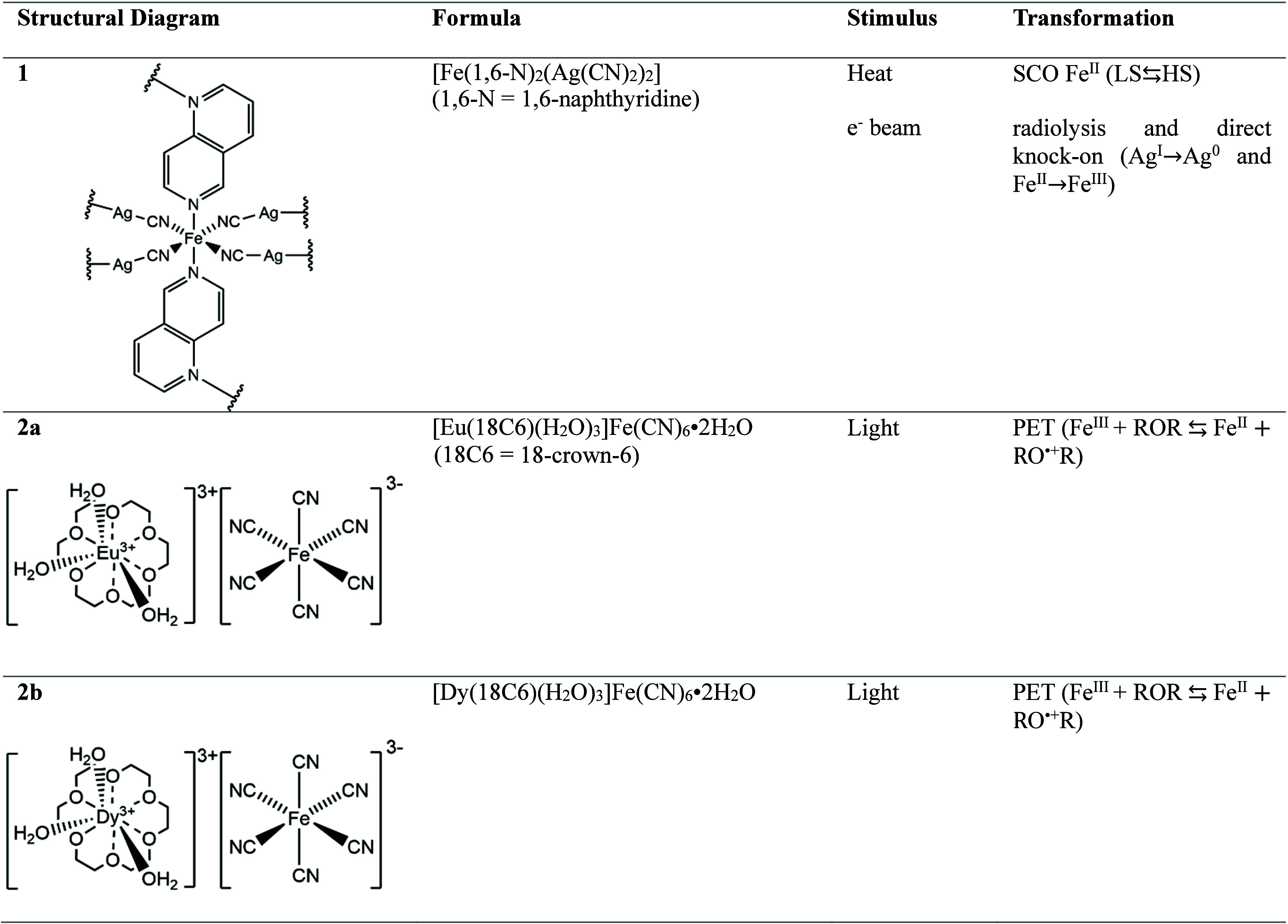
Compound Nomenclature, Structural Diagrams, Formulas and Stimuli Required to Initiate Spin-State Switching Alongside the Type of Chemical Transformation^a^

a Crystallization water molecules in schematic diagrams for compounds **2a** and **b** have been omitted for clarity. PET = photoinduced electron transfer; SCO = spin crossover.

## Results and Discussion

### Heat-Induced Spin-Crossover of Compound **1**

**Figure 2:**
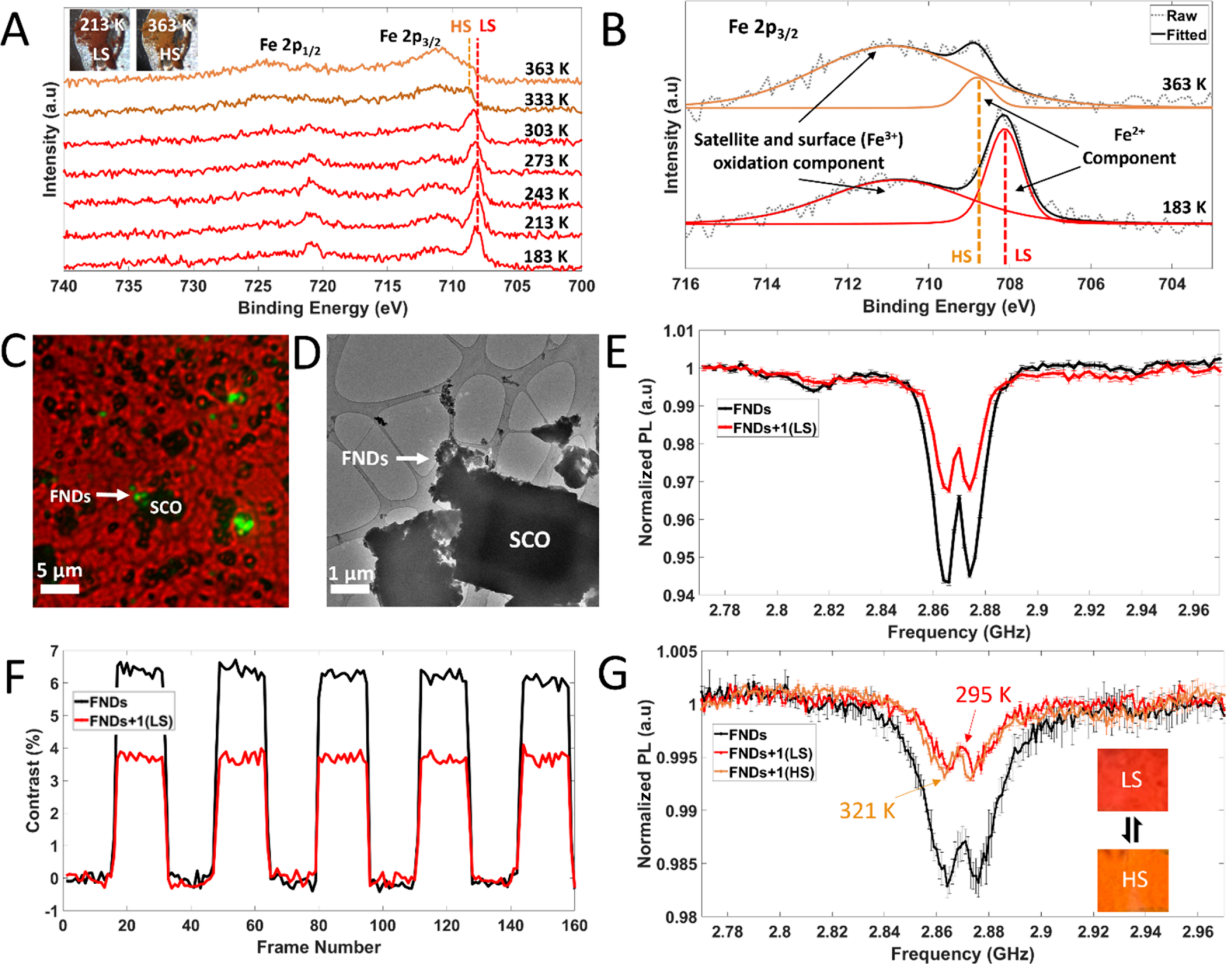
(A) Variable-temperature XPS Fe(II) 2p photoelectron spectra for compound **1** showing surface chemical changes upon SCO. (B) A high-resolution XPS spectrum of the Fe 2p_3/2_ region at 363 and 183 K, showing HS and LS photoelectron behavior, respectively. Transition from the LS to HS state corresponds to a greater contribution of the satellite component. (C) Overlayed bright-field and fluorescence optical image, FNDs are shown as bright green spots. (D) TEM image showing a single microparticle of SCO compound **1** in direct contact with a small FND cluster over the hole of the carbon film. (E, F) Room-temperature ODMR and MM NV^–^ sensing, respectively, from the FND cluster highlighted with a white arrow in panels (C) and (D)). Upon addition of **1** (in the LS state) ODMR and MM contrast is reduced. (G) ODMR of **1** in both the LS (295 K) and HS (321 K) states. The NV^–^ resonant frequency was used to estimate temperature which was compared against a thermocouple attached to the GCS (thermocouple reading 328 K). Inset shows photographs of a section of the powder on the GCS at both 295 and 321 K (red low- spin and orange high spin, respectively). Singal to noise in panel (G) was lower due to the lower NA air-coupled objective used in heating measurements.

The recent study by Lamichhane et al. employing NV magnetometry to study an Fe^II^ SCO compound showed that the LS state (theoretically diamagnetic in the bulk) showed paramagnetic behavior, potentially due to surface oxidation^18^ or surface Fe atoms in the HS state (present at all temperatures) due to partial Fe coordination.^23^ We set out to sense switchability (detect both LS and HS states) of an Fe^II^ SCO complex (magnetic hysteresis centered at room temperature)^4^ using an in situ heating NV^–^ sensing methodology. Microcrystalline powder of compound **1** was synthesized using a previously reported method yielding irregular micrometer-sized cuboid particulates (0.3–5.7 μm, TEM analysis; see Figure S1).^4^ The powder X-ray diffraction (PXRD) pattern of **1** was consistent with the original reports from Hiiuk et al. confirming phase purity (see Figure S2). Prior to NV^–^ sensing measurements, the SCO surface in **1** was characterized by variable-temperature XPS (Figure 2A). XPS probes changes in chemistry at depths of up to 10 nm, similar to the paramagnetic detection range of FND particles using our previously reported methodology,^22^ thereby it provides a useful profile of the paramagnetic environments which possess fluctuating spin noise that will couple to NV^–^ centers in FND particles through dipole–dipole coupling. As seen from the Fe 2p_3/2_ region, there are significant changes in photoelectron lines upon SCO (Figure 2B). The Fe^II^(HS) state has longer bonds around the Fe coordination sphere, hence weaker electron ligand-to-metal-charge transfer (LMCT), which results in a greater effective nuclear charge experienced by the Fe 2p electrons, therefore the binding energy of the Fe 2p electrons increase. Unpaired 3d electrons in the HS state also have an increased degree of spin–orbit coupling to ejected photoelectrons, leading to a larger satellite component, as observed.^24,25^ XPS measurements therefore are sensitive to changes in the chemical environment of Fe^II^ upon SCO in **1**.

Initial NV^–^ sensing optically detected magnetic resonance (ODMR) and magnetic modulation (MM) measurements, on TEM grids (Figures 2C and 2D for correlative light-electron microscopy (CLEM) imaging), of **1** showed paramagnetic behavior in the LS state, in agreement with measurements by Lamichhane et al., evidenced by ODMR and MM contrast reduction (Figures 2E and 2F, respectively), from spin-noise induced NV^–^ depolarization.^22,26−28^ To test SCO switching detectability, in situ heating was employed (Figure 2G). First, the LS state of **1** was measured at room temperature (293 K) on a FND functionalized glass coverslip (GCS), which gave a large paramagnetic ODMR response due to surface Fe oxidation (Fe^III^) and/or the presence of surface HS state Fe atoms in the bulk LS state temperature regime. The GCS containing drop-cast **1** was then heated in a furnace to 353 K, where a thermochromic color change from red to orange was observed, indicative of a bulk SCO switch (inset of Figure 2G). The sample was then transferred to a heating stage on the microscope that maintained the internal stage temperature at ∼321–328 K during ODMR measurements, above the SCO transition (hysteresis centered at 313 K). Temperature was monitored using both the temperature-dependent microwave resonant frequency shift of nanodiamonds (321 K),^29^ as well as a thermocouple affixed to the glass slide (328 K). Upon heating, no change in ODMR contrast was observed. We believe this is due to the surface sensitivity of our ODMR measurements: the presence of Fe^III^ due to surface oxidation and surface HS state Fe for the sample in both bulk LS and HS states, which dominate ODMR contrast changes. The bulk of the microparticles of **1** will undergo switching LS ⇆ HS in a SCO process, but as NV^–^ dipole–dipole interaction strength varies with the inverse cube of distance (*r*^–3^),^30^ predominant dipole–dipole interactions will occur from surface spins, i.e., paramagnetic oxidized Fe^III^ and surface HS state Fe centers.

**Figure 3:**
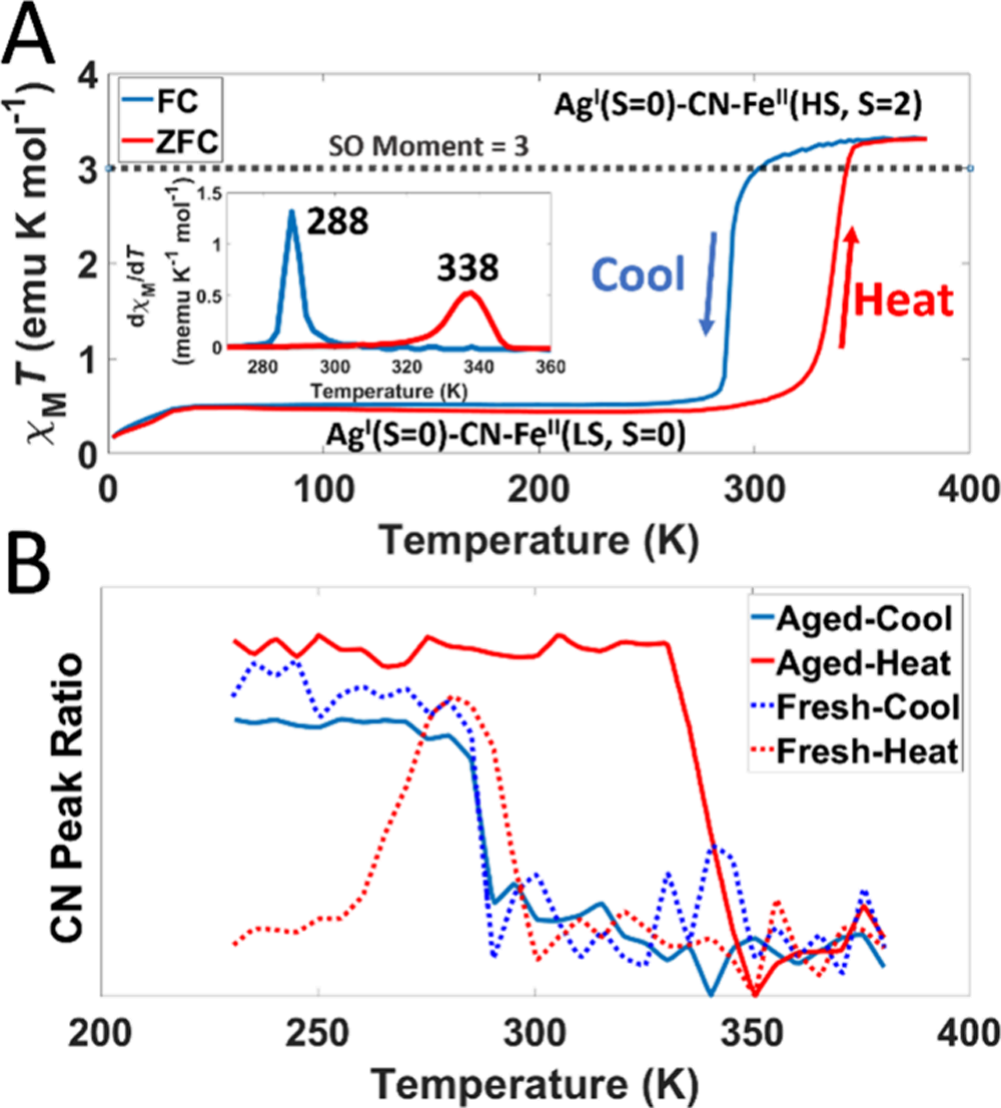
(A) Thermal variation of **χ**_**m**_***T*** for “aged” compound **1** showing hysteretic switching between LS (diamagnetic) and HS (paramagnetic) spin states. Field-cooled and zero-field-cooled susceptibility indicate the cooling and heating cycles, respectively. (“Aged” powder, SQUID: LS → HS at 338 K, HS → LS at 288 K). The spin-only (SO) magnetic moment for an Fe(II)-HS center is shown by the dashed black line (3 emu K mol^–1^). Inset: FC ZFC dχ/d*T*(*T*). (B) Raman C≡N band ratio analysis between LS and HS states (Figure S4 for full Raman spectra and more information on C≡N band ratio calculation) for “fresh” and “aged” powders of compound **1** illustrating the structural changes accompanying SCO. (“Aged” powder, Raman: LS → HS at 338 K, HS → LS at 287 K. “Fresh” Powder, Raman: LS → HS at 294 K, HS → LS at 287 K.)

SCO in the bulk was confirmed by SQUID magnetometry and Raman spectroscopy measurements (Figure 3A and S7, respectively). At temperatures below the SCO transition, χ_M_*T* is close to 0 emu K mol^–1^, consistent with the majority of metal centers being in diamagnetic states, i.e., Fe^II^(LS) and Ag^I^. χ_M_*T* increases rapidly through the SCO transition to a value consistent with HS Fe^II^ with *S* = 2 and unquenched orbital angular momentum due to the ^5^T_2g_ ground state (χ_M_*T* = 3.3 emu K mol^–1^). Field-cooled (FC) and zero-field-cooled (ZFC) susceptibility differed from data previously reported for compound **1**.^4^ We observed a larger temperature hysteresis (288-338 K, cf. 283–293 K)^4^ centered at a higher temperature (313 K, cf. 288 K)^4^ (Figure 3A).

Interestingly, Blanco et al. found that surface oxidation of Fe^II^ SCO compounds can alter the magnetic hysteresis, with the presence of an oxidized surface stabilizing and prolonging the LS → HS transition.^31^ Indeed, the presence of a finite susceptibility at temperatures below SCO suggests there may be paramagnetic surface impurities (Figure S3).^23^ Therefore, we investigated the effect of air exposure by comparing the properties of samples exposed to air for >4 days at room temperature, “aged”, and just after synthesis (also in air), “fresh”. We found that, under ambient conditions, “fresh” samples were orange, indicating a HS state, but “aged” samples were red, indicating a LS state, and required heating to higher temperatures to revert to the orange HS state. This was quantitatively borne out through variable-temperature Raman spectroscopy, where the C≡N bond intensity ratios are a sensitive indicator of the spin state (Figure 3B, as well as Figure S4 for full Raman spectra). “Fresh” powders (synthesized the same day as measurement) gave a hysteretic response in Raman spectroscopy temperature profiles with temperatures close to reports by Hiiuk et al., whereas the behavior of “aged” powders closely matched SQUID measurements herein (Figure S5). “Fresh” powders also showed greater reversibility in the Raman spectra, with the “aged” powder not seemingly able to fully retain the initial LS C≡N band ratio, indicating an irreversible structural change. Thermogravimetric analysis (TGA) of “aged” powders in air only showed a structural decomposition onset at 398 K (corresponding to 1,6-naphthrydine loss), confirming no heat induced chemical decomposition during SQUID magnetometry and Raman spectroscopy measurements (Figure S6). In an attempt to quantify the extent of surface oxidation in “aged” samples, Ar^+^ etching to a depth of ∼100 nm into single crystals of **1** was performed (Figure S7). Upon etching, the XPS spectrum showed a shoulder component in the Fe^II^ region, potentially indicating removal of surface Fe^III^ oxidation in the crystal. However, upon etching, loss features in the Ag 3d region appeared, indicating the formation of Ag^0^ metal, via site-selective degradation of **1**.^32^

Overall, these measurements indicate that the surface of compound **1** undergoes oxidation under ambient conditions, resulting in paramagnetic Fe^III^. These species, as well as any surface HS state Fe ions, make detection of the thermally induced Fe^II^ LS⇆HS switching by FND NV^–^ sensing methods used herein, not immediately feasible under ambient conditions. Because of the nature and range (ca. 30 nm) of this NV^–^sensing, if one is to study bulk switching phenomena, it is important that the material of interest does not have a chemically separate spin-active surface which could obscure sensing of target magnetic switching mechanisms. However, this technique could be advantageous if detection of surface magnetic spins is of particular research interest.

### Electron-Beam-Induced Structural and Magnetic Changes in Compound **1**

**Figure 4:**
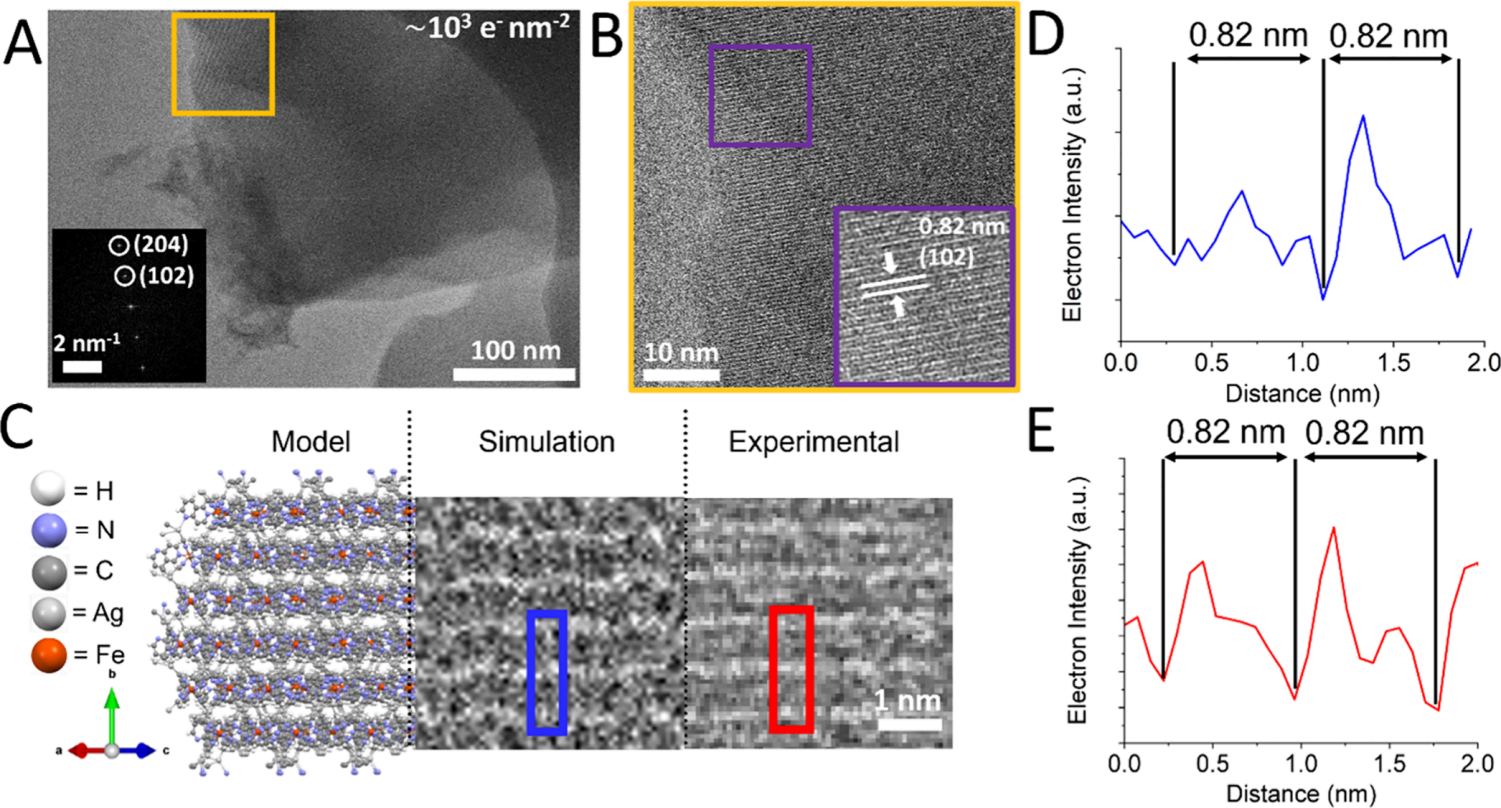
(A) HRTEM image at low e^–^ beam fluence (∼10^3^ e^–^ nm^–2^) of a thin area of a microparticle of compound **1**. Inset of (A) shows a fast Fourier transform (FFT) image of the area marked in orange, first- and second-order diffraction is identified for the (102) and (204) planes, respectively. (B) Zoomed-in orange area shown in panel (A). Lattice fringes in HRTEM images correspond to the (102) planes (*d*-spacing is identified, marked with arrows and indexed). (C) A molecular model (left), simulated TEM image (middle) and experimental HRTEM image (right) of the (102) plane. (D, E) Line profiles of the simulated (blue box and trace) and experimental image (red box and trace), respectively.

**Figure 5:**
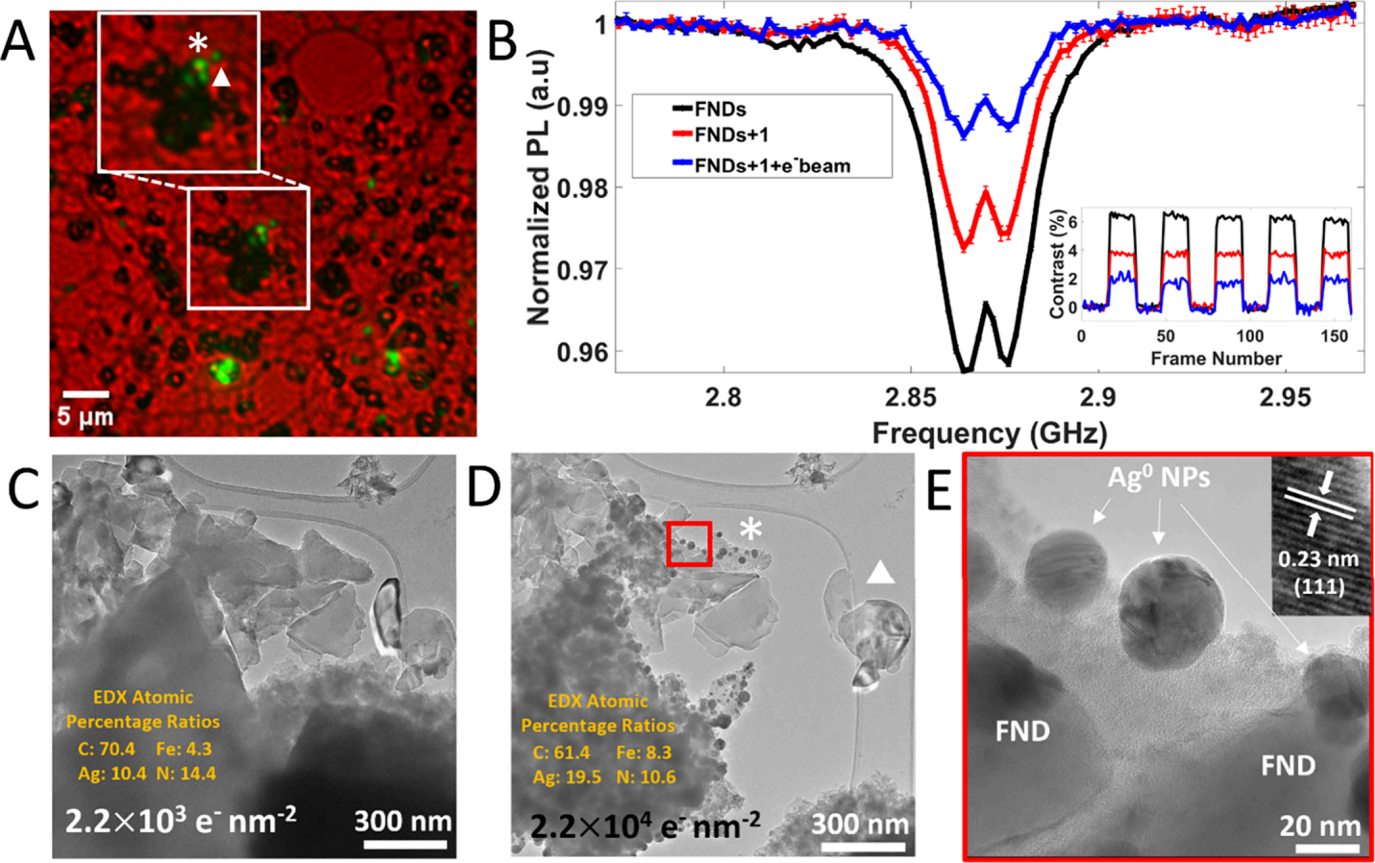
(A) Overlayed bright-field and fluorescence images before (white square) e^–^ beam irradiation, indicating the FND cluster chosen for analysis (white asterisk) and the interacting particles of compound **1**. (B) ODMR spectra and MM traces taken from the target FND cluster (labeled in panel (A) as a white asterisk) taken before and after the addition of compound **1**, as well as after e^–^ beam irradiation of the microcube. (C) TEM image of the interacting microparticle of **1** and the FND cluster at low e^–^ fluence (stated at the bottom of images). (D) TEM image of the interacting microcube and FND cluster at higher e^–^ fluence (the FND cluster labeled with a white triangle illustrates the distance relationship between clusters shown in panel (A)). At relatively moderate flux (>10^3^ e nm^–2^ s^–1^), we observed the beam sensitivity of compound **1**. Insets of panels (C) and (D) also show EDX atomic percentage ratios taken for pristine and irradiated particles, respectively (Figure S13 for EDX spectra). (E) HR-TEM image from the area marked in panel (D) (red square) showing Ag^0^ metal NPs formed as a product of e^–^ beam-induced transformations on the surface of the SCO cube. Inset of (E) shows a common *d*-spacing found on the Ag^0^ NPs, shown to correspond to the (111) plane. Note as a control measure: At the e^–^ fluence used to induce transformations in compound **1**, the sensing properties of the NV^–^ centers within FND particles remained unchanged (Figure S23).

Compounds **1** and **2** contain e^–^ beam sensitive organic ligands. e^–^ beam irradiation at significant electron fluence will chemically alter the state of matter through a number of different damage mechanisms.^8,33^ Upon imaging compound **1**, e^–^ beam sensitivity was observed. We set out to see if we could monitor the magnetic changes of a material using our finder grid NV^–^ sensing methodology induced by e^–^ beam transformations. High-resolution TEM (HRTEM) imaging of individual particles of compound **1** at low e^–^ fluence (∼10^3^ e^–^ nm^–2^) revealed lattice fringes which matched the (102) planes spacing, *d* = 0.82 nm, identified in bulk PXRD measurements (see Figures 4A and 4B). TEM experimental and simulated images alongside molecular modeling of the (102) planes are shown in Figure 4C, with line profiles for simulated and experimental images shown in Figures 4D and 4E, respectively. Drop casting microparticles of “aged” samples of **1** and FNDs onto a TEM grid yielded FNDs in close proximity (direct contact or less than ∼30 nm) to the microparticles (Figure 5C). Using the methodology previously demonstrated by Flinn et al.,^22^ we can measure NV^–^ response of spin-active particles before and after e^–^ beam irradiation, without the e^–^ beam changing NV^–^ sensing contrast by employing low e^–^ fluence conditions. The effects of the 200 keV e^–^ beam on NV^–^sensing contrast at varying e^–^ fluence was measured. Significant reduction in contrast above experimental error was only observed at e^–^ fluence of >5 × 10^4^ e^–^ nm^–2^ (Figure S8). Plots of diffraction spot intensity as a function of cumulative e^–^ fluence, in selected area electron diffraction (SAED) patterns, suggested translation and rotation of FND crystals as diffraction spot intensities fluctuate during irradiation, as opposed to intensity changes due to e^–^-beam-induced structural damage (Figure S9).^34,35^

As particles of **1** are e^–^ beam sensitive, the e^–^ beam even at low fluence can impose changes to the structure **1**, as an insulating metal–organic framework (MOF), will be particularly sensitive to electron–electron interactions via radiolysis (ionization), heating and direct knock-on (DKO), which can break chemical bonds forming different structures.^36^ Upon e^–^ beam irradiation (>10^3^ e^–^ nm^–2^) Ag^0^ nanoparticles (NPs)/rods form in **1** (see the time series video file in the Supporting Information) in close proximity to FNDs (Figure 5D and 5E, see Figures 5A and 5C for CLEM imaging). NV^–^ sensing was conducted at two different low e^–^ beam fluence conditions: 2.2 × 10^3^ and 2.2 × 10^4^ e^–^ nm^–2^, respectively. We found ODMR and MM contrast to be reduced when increasing e^–^ beam fluence (Figure 5B) for FND clusters in close proximity to particles of **1** (results from a different grid location can be seen in Figure S10). Before e^–^ beam irradiation, we conclude that a decrease in contrast is observed from surface oxidation (Fe^III^) and surface HS state Fe paramagnetic centers, previously identified by Lamichhane et al.^18^ and Coronado et al.^23^ Upon e^–^ beam irradiation, ionization of Fe^II^ → Fe^III^ caused by radiolysis triggers chain-like secondary electron damage within the MOF structure of **1**.^36^ The excited electron–lattice system proposed, induces the reduction of diamagnetic Ag^I^ to Ag^0^ (Figure S11 for a full discussion of the proposed transformation mechanism). In the pristine state, Ag^I^ centers within the structure, have strong argentophilic interactions prior to e^–^-beam-induced transformations, therefore Ag atoms are already in a preinteracting state.^4^ Electron fluence was gradually increased and EDX ratios (atomic percentage shows as an inset of Figures 5C and 5D for pristine and modified by e^–^ beam states of **1**, respectively) confirmed the mechanism leading to Ag^0^ NP and rod growth. As a control measurement, Ag^0^ NPs synthesized by a previously reported method,^37^ were drop cast on a GCS and a TEM finder grid, and resulted in a reduction in NV^–^ ODMR and MM contrast (Figure S12). Metallic Ag can induce a range of effects on NV^–^ spin coherence, including reduction of NV^–^ longitudinal spin relaxation times, *T*_1_, for example through magnetic Johnson noise emanating from conducting Ag films.^38^ This suggests that low levels of magnetic noise can perturb NV^–^ sensing properties. We draw the corollary that, in the presence of Ag^0^ NPs, the efficiency of optical polarization of NV^–^ spins to the *m*_s_ = 0 ground state will be less efficient, potentially explaining the reduction in ODMR and MM contrast observed upon Ag^0^ NP addition and e^–^ beam irradiation.^15,27,39^

e^–^ beam-induced enhancement of spin noise may also occur through the ionization of bulk diamagnetic Fe^II^ (*S* = 0) to paramagnetic Fe^III^ (*S* = ^1^/_2_ or ^5^/_2_) following the proposed e^–^ beam ionization mechanism. Electron energy loss spectroscopy (EELS) of pristine and e^–^ beam-modified particles was used to investigate the oxidation state of Fe monitoring the L_3_ loss edge (Figure S13). For the e^–^ beam-modified state, there is an increase in the ratio of higher to lower energy loss peaks, corresponding to a greater amount of Fe^III^, consistent with the proposed reaction mechanism.^40^ Also, for the e^–^ beam-modified state, the EELS N K-edge diminished into spectral noise, consistent with loss of nitrogen through HCN gas. Retention of carbon and nitrogen in the 1,6-N ligand is expected for amorphous aromatic material under e^–^ beam irradiation, similar to previous reports.^34^ The chemical structure of the remaining metallic Fe-1,6 naphthyridine network after Ag^0^ formation is unknown due to the rapid loss of crystallinity.

Overall, changes in the structure of **1** induced by the e^–^ beam led to a reduction in ODMR and MM contrast produced by a combination of e^–^-beam-generated metallic Ag^0^ NPs and paramagnetic Fe^III^ ions, following the concentration-dependent dipole–dipole interaction between spin-active species and NV^–^ centers.^13^ This correlative FND sensing methodology, allows one to observe magnetic changes in a single particle of a material upon irreversible transformations caused by e^–^ beam irradiation.

### Photomagnetism—Bulk Measurements in Compound **2a**

**Figure 6:**
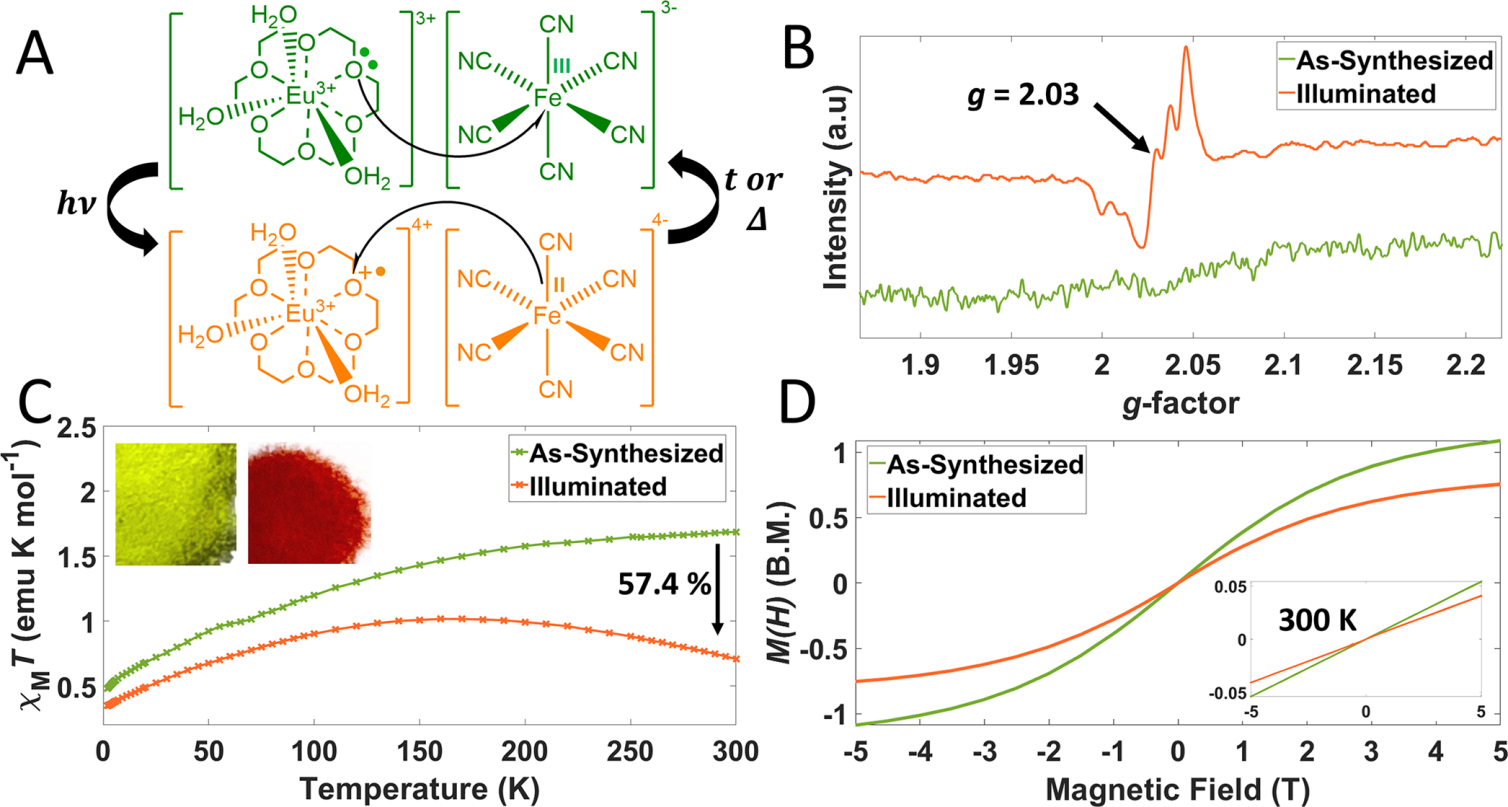
(A) Mechanism of reversible PET. Light (*hv*) is used to initiate the forward transformation of the powder, whereas either time (*t*, >1 week) at room temperature, or heat (Δ, 80 °C for 2 h) can be used to reverse the phototransformation. (B) Solid-state EPR spectra of as-synthesized and illuminated powders of **2a**. (C) Thermal variation of the value **χ**_**m**_***T*** for compound **2a** in both the as-synthesized (green-yellow) and illuminated state (orange). Photodemagnetization at 300 K gives a change in susceptibility of 57.4%. Inset photographs show powders of **2a** in both states. (D) *M*(*H*) curve for the as-synthesized and illuminated states of **2a** at 2 K. Inset shows *M*(*H*) at 300 K.

Compound **2a** was synthesized by a previously reported method, which yielded microcrystalline powders (1.0–20.9 μm; see the TEM analysis in Figure S1). Compound **2a** is photomagnetic with Fe^III^ → Fe^II^ photo switching, thereby mitigating surface oxidation (seen with compound **1**) by starting with Fe in a higher oxidation number. **2a** is photochromic and, upon illumination (456 nm), a yellow-green to orange color change following the photoinduced electron transfer (PET) mechanism (Figure 6A) was observed.^5^ Fourier transform infrared (FTIR) spectroscopy confirms significant changes in structure upon illumination, with large changes in the C≡N and crown ether C–O–C absorbance stretches observed (Figure S14).^5^ PXRD of both as-synthesized and illuminated states of **2a** matched the reports of Cai et al. confirming the phase purity and retention of crystallinity upon illumination. To confirm the expected photomagnetic properties, SQUID magnetometry was performed. χ_M_*T* vs temperature (Figure 6C), alongside magnetization vs field, *M*(*H*), measurements (Figure 6D), both at 2 and 300 K were conducted. χ_M_*T* measurements gave a photodemagnetization at 300 K susceptibility of 57.4%, compared to 33.5% reported by Cai et al. A greater change in χ_M_*T* upon illumination indicates a greater extent of photoconversion (see IR spectra discussion in Figure S14). *M*(*H*) measured at 2 K approaches the saturation magnetization (*M*_sat_) of 1 μ_B_, indicative of one unpaired electron, consistent with Fe^III^(LS), *S* = ^1^/_2_ and Eu^III^ in the ^7^F_0_ ground state. Upon illumination, *M*_sat_ reduces, which is consistent with the previously described antiferromagnetic interactions between oxygen radicals and europium.^5^*M*(*H*) at 300 K does not saturate for as-synthesized or illuminated powders, indicating the presence of antiferromagnetic interactions in the room-temperature paramagnetic regime. EPR spectroscopy found negligible response for as-synthesized green powders of **2a** due to the presence of significant spin–orbit coupling,^41^ and fast spin–lattice relaxation.^42^ Upon illumination, a signal appeared at *g* = 2.03, consistent with prior observations by Cai et al. (see Figure 6B).^5^ Similar EPR profiles of Eu complexes have been reported.^5,43^

### Photomagnetism—Surface Measurements in Compounds **2a** and **2b**

**Figure 7:**
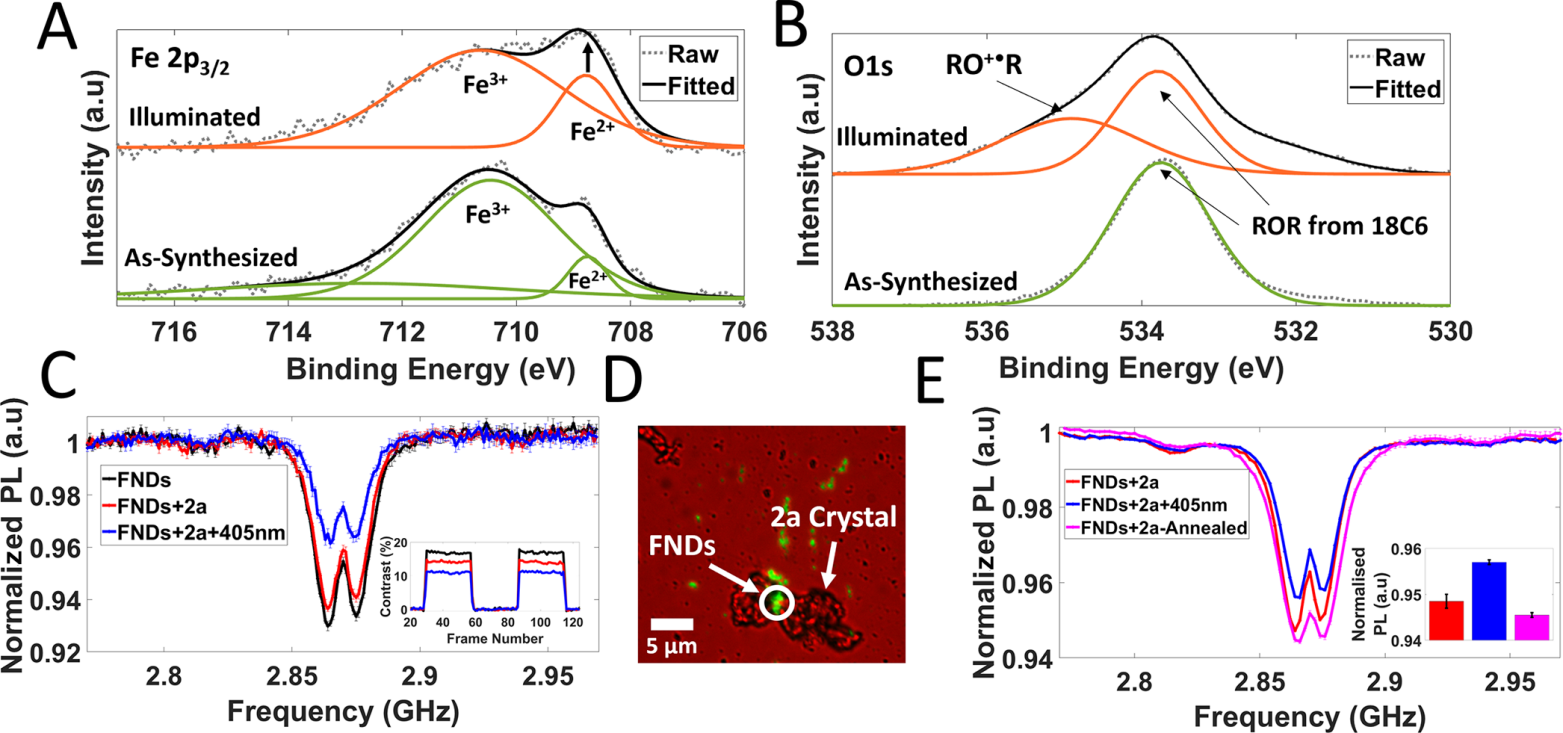
(A, B) High-resolution XPS spectra of the Fe 2p_3/2_ region (panel (A)) and O 1s region (panel (B)) for as-synthesized and illuminated states of **2a**. (C) ODMR spectra (inset show MM traces) for the FND cluster highlighted in panel (D) by a white circle, including illuminated states by 405 nm light (blue traces) on a GCS. (D) Overlayed bright-field and fluorescence image showing an example of a crystal of **2a** that is in contact/close proximity to clusters of FNDs on a glass coverslip. (E) ODMR spectra probing the reversibility of the photoinduced electron transfer mechanism of **2a**, including an annealing step to reverse the phototransformation. Set of measurements for (E) were conducted on TEM finder grids for ease of location after annealing. Inset of panel (E) shows the averaged normalized PL ODMR double minima values from spectra in panel (E) as a bar chart.

To investigate the presence of photomagnetism at the surface of particles of **2a**, XPS analysis was conducted for both as-synthesized and illuminated crystalline powders. High-resolution spectra for the O, Fe, and Eu regions show evidence of surface PET. Wide-scan spectra confirm the presence of all expected elements (Figure S15). For as-synthesized **2a**, the Fe 2p_3/2_ region (Figure 7A) consisted mainly of Fe^III^ signal (710.4 eV) with a small shoulder (708.7 eV), corresponding to Fe^II^, potentially indicating partial phototransformation from ambient light conditions.^44^ The O 1s region (Figure 7B) consisted of only one environment (533.7 eV). Upon illumination, the ratio of Fe^II^ signal compared to Fe^III^ increased and a second oxygen environment at higher binding appeared (534.9 eV) emerged, likely corresponding to the photogenerated oxygen radical cation (RO^+ •^R), formed by electron transfer from crown oxygen PET to Fe^III^ centers. The Eu 3d spectrum (Figure S16) shows evidence of negligible changes in Eu oxidation state upon illumination, in agreement with the PET mechanism proposed by Cai et al. Therefore, surface and bulk spectroscopy agree and show feasibility for NV^–^ sensing measurements of the switchable spin states.

The intensity and spectral profile of the Eu^III^ center in **2a**, in the NV^–^ detection range (575–800 nm), were investigated via PL emission spectroscopy. PL collected from a 100 μm × 100 μm field of view centered at typical target sites showed that the Eu^III^ PL signal is considerably low against NV PL emission signal (Figure S17). Low Eu^III^ PL could be from weak magnetic superexchange interactions between Eu and Fe through the cyano bridge, which quenches Eu emission.^45^ Room-temperature NV^–^ sensing measurements of as-synthesized crystals drop-cast on GCS functionalized with FNDs (Figure 7D) showed small decreases of NV^–^ PL contrast due to dipole–dipole interactions between NV^–^ centers and paramagnetic Fe^III^ and Eu^III^ centers (Figure 7C). Thermal population of Eu^III^ magnetic energy levels above the diamagnetic ground state will occur at room temperature. Upon illumination of **2a** crystals by blue light (405 nm from the objective), a comparatively large contrast reduction in both the ODMR spectra and MM traces was observed (Figure 7C), indicative of increased spin-noise signal. This corresponds with a clear green-yellow to orange color change of **2a** on the GCS. Bulk SQUID measurements show a reduced magnetic susceptibility value at room temperature for illuminated species due to antiferromagnetic interactions between the crown ether radical and Eu^III^ centers. Upon illumination, the number of unpaired electrons does not change, one electron from the lone pair on crown ether oxygen atoms is transferred to an Fe^III^-LS center (one unpaired electron in the octahedral Fe T_2g_ set of atomic orbitals), creating a radical cation on the oxygen center (again leaving one unpaired electron in the 2p orbitals, i.e., *S* = ^1^/_2_ is retained). The reduced ODMR and MM contrast upon light illumination of **2a** could originate from many sources. The movement of a largely orbital dominant moment in Fe^III^^41^ to the spin RO^+ •^R radical, as well as the room-temperature moment on the Eu^III^ ion interacting with the RO^+ •^R radical could affect the NV^–^ sensing contrast. Moreover, evidence for hyperfine structure in the EPR spectrum of the illuminated state (Figure 6B), observed for similar systems in previous reports,^43,46^ suggests coupling between the RO^+ •^R radical and spin active nuclei, such as ^151^Eu (47.8%) and ^153^Eu (52.2% abundance) both with ^5^/_2_ nuclear spin, which could increase the importance of the nuclear spin-bath interactions with NV^–^ centers.^47,48^ Although the detailed mechanism underlying photoinduced contrast reduction remains unclear, our conjecture is analogous to spin-noise mediated reduction in NV^–^ relaxation times reported for other chemical systems in the literature.^28,49,50^ Contrast reduction was also shown to be dependent on the illumination time (Figure S18), similar to the evolution of the IR spectrum observed by Cai et al.^5^ To probe the reversibility of this phototransformation, illuminated microcrystals of **2a** on a TEM finder grid were annealed (2 h at 80 °C on a hot plate in an Al-foil-lined Petri dish in air).^5^ ODMR contrast recovery was observed for the annealed state as expected; however, contrast recovery surpassed the initial “as-synthesized” state, potentially indicating an incomplete transformation. Greater transmittance intensity (%) in the IR spectrum of material **2b** for the Fe^II^C≡N band in the “recovered” state, compared to the “as-synthesized” state, was observed for Cai et al., indicating incomplete reverse structural transformations.^6^

NV^–^ sensing control measurements conducted on bare FNDs, as well as Eu(NO)_3_, K_3_Fe(CN)_6_ or 18C6 (precursor components of **2a**, Figure S19) showed no change in sensing contrast upon the same illumination conditions, confirming the change in contrast to be from the target PET mechanism. NV^–^ sensing experiments were also conducted in the liquid phase where crystals of compound **2a** were dissolved in water (1 mg mL^–1^) and pipetted (200 μL) onto a GCS functionalized with FNDs. After 10 min of 405 nm illumination from the objective, the same trends were observed as for solid-state measurements, suggesting the PET mechanism between aqueous cationic [Eu(18C6)(H_2_O)_3_]^3+^ and [Fe(CN)_6_]^3–^ anions persists (Figure S20). The same color change in solution as seen in the solid state was observed, i.e., yellow-green to orange.

Similar NV^–^ in FND sensing results, ODMR and MM contrast reduction upon illumination, were also observed for the Dy analogue (**2b**, Figure S21). This analogue has the opposite bulk trend (compared to **2a**) upon illumination, showing an increase in magnetic susceptibility (10.0%, compared to 20.9% reported by Cai et al., Figure S22) suggested to be due to ferromagnetic-like interactions being present at room temperature, following the same PET transfer mechanism.^6^ Eu and Dy analogues both show the same surface trend in paramagnetic NV^–^ response upon illumination, although having different types of bulk magnetic interactions (antiferromagnetic and ferromagnetic respectively as seen from SQUID data). The results herein show that reversible surface photomagnetism can be detected by the NV^–^ sensing methodology using FNDs, a step toward quantum technological applications based on diamond defect sensing.

## Conclusions

We show that NV^–^ centers in FND particles can be used as nanoscale probes for sensing surface magnetic switchability across a range of stimuli, specifically light illumination and e^–^ beam irradiation, in recently developed room-temperature magnetic switching cyanometallic frameworks. We have employed a nonintegrated CLEM NV^–^ sensing platform, on TEM finder grids, to observe phenomena down to the single-particle level. This surface sensitive methodology allowed us to observe that SCO particles of material **1** had surface oxidation (presence of paramagnetic Fe^III^ in both the LS and HS states), which obscured the detectability of NV^–^ sensing, in the form of ODMR and MM, to study Fe^II^ LS ⇆ HS switching, which is routinely observed in bulk characterization. This demonstrates the importance of studying switchable magnetic materials both in the bulk and on the surface, especially as surface properties become important for nanomaterials with a high surface-to-volume ratio. “Fresh” and “aged” powders of SCO material showed different hysteretic properties, evidenced by SQUID and Raman spectroscopy, due to the oxidized surface stabilizing and prolonging the LS → HS transition. We propose if one is to study bulk magnetic switching via similar NV^–^ sensing methods, the material of interest should not have a chemically separate spin-active surface, which could obscure the sensing of target magnetic switching mechanisms. Irradiation with a 200 keV e^–^ beam triggered chemical transformation in single, well-defined particles of **1**, which were identified as ionization of Fe^II^ centers to Fe^III^ and reduction of Ag^I^ to Ag^0^, based on HRTEM images, EDX and EEL spectroscopic analysis. NV^–^ centers in FNDs were able to detect the changes in spin noise induced by e^–^ beam irradiation in the MOF. These changes are irreversible and associated with significant structural modifications in the particle of **1**.

Photomagnetic lanthanide (Eu and Dy) crown ether hexacyanoferrate single-molecule magnets proved to be ideal candidates for switchable single-particle magnetic sensing. XPS analysis revealed chemical changes upon illumination consistent with the PET process Fe^III^ + (18-crown-6) ⇆ Fe^II^ + (18-crown-6)^+ •^. Interactions of NV^–^ with switchable spin-noise on the surface of microparticles of compounds **2a** and **2b** offers an effective mechanism for NV^–^ sensing using FNDs. Importantly, the photoexcited states of **2a** and **2b** can be reversed back to the ground states, activated thermally, thus ensuring reversibility of the magnetic switching.

Miniaturization of the spin detection volume as well as the spin sensor itself is key to many quantum technology applications including spintronics and molecular switching nanodevices. The example of reversible magnetic switching triggered by light and sensed by NV^–^ centers implanted in FNDs at the single-particle level could have future applications in nanoscale magnetic information storage and processing devices.

## Experimental Section

### Materials

Carboxylated fluorescent nanodiamonds (FNDs) were purchased from FND Biotech, Inc. (brFND-100). The average FND diameter was 100 nm, containing >1000 NV centers per FND particle.^51^ Prior to NV^–^ sensing, FNDs were either dried onto a glass coverslip (GCS) from a 0.1–0.5 mg mL^–1^ suspension and then incubated at 60 °C for at least 12 h. or drop-cast onto TEM grids (dried in air for at least 4 h), for identical-location correlative light-electron microscopy measurements. Compounds Eu(NO_3_)_3_·5H_2_O, DyCl_3_·6H_2_O, *p*-toluenesulfonic acid monohydrate, tannic acid, AgNO_3_, trisodium citrate, 18-crown-6, and K[Ag(CN)_2_] were purchased from Sigma–Aldrich, K_3_Fe(CN)_6_ and dimethylformamide from Fisher Scientific, 1,6-naphthyridine (from ABCR UK), and Fe powder (from Strem Chemicals). All chemicals were used as received without further purification.

#### Fe Ag Cyanoheterometallic [Fe(1,6-N)_2_(Ag(CN)_2_)_2_] – 1,6-N = 1,6-naphthyridine (**1**)

Powdered and crystalline samples were prepared following a previously reported method.^4^ Raman bands (cm^–1^): 2098 and 2158 (C≡N).

### Lanthanide Crown Ether Hexacyanoferrate

**[X(18C6)(H**_**2**_**O)**_**3**_**]Fe(CN)**_**6**_**·2H**_**2**_**O**, where 18C6 = 18-crown-6 and X = Eu or Dy (**2a** or **2b**, respectively). Microcrystalline powders were synthesized by a two-step process. First, a precursor [X(DMF)_4_(H_2_O)_3_(μ-CN)Fe(CN)_5_]·2H_2_O (DMF = dimethylformamide, X = Eu or Dy) were synthesized by a previously reported method.^52^ These were then added to a solution containing 18-crown-6 to obtain the target compound via the reported procedure.^53^**2a**: Elemental microanalysis found: C 29.7, H 4.6, N 11.5% (calculated: C 30.1, H 4.8, N 11.7%) w/w. ATR-IR (ν max cm^–1^): 2122 and 2149 (C ≡ N). **2b** Elemental microanalysis found: C 28.6, H 5.02, N 11.1% (calculated: C 29.7, H 4.67, N 11.5%) w/w. ATR-IR (ν max cm^–1^): 2124 and 2155 (C ≡ N).

### Ag^0^ Nanoparticles

Silver NPs were synthesized adapting a previously reported procedure.^37^ A typical synthesis involved: 10 mL of 6.8 mM aqueous trisodium citrate was added to 10 mL of 23.5 μM tannic acid and heated to 60 °C. This solution was then added to 80 mL of 0.74 mM AgNO_3_ (also preheated to 60 °C) with vigorous stirring. Immediately, upon addition, a typical yellow-brown color was observed indicating the formation of Ag^0^ NPs. The mixture was then boiled for 30 min, cooled down to room temperature and stored in a foiled lined flask at 3 °C.

### Nitrogen-Vacancy Paramagnetic Sensing

NV^–^ sensing protocols were adapted from a previously reported method.^22^ For ODMR studies the microwave frequency was swept from 2.77 to 2.97 GHz in 1 or 2 MHz steps to probe the ground state NV^–^ transition. In MM the off axis magnetic field was applied via an electromagnet placed in close proximity to the samples at a frequency between 50 and 500 mHz to modulate applied magnetic field strength between 0 and 40 mT. Image frame exposure time: 30 ms for GCS and 100–307 ms for TEM grids. For *in situ* heating experiments the light was focused onto the back focal plane of an air coupled 20× objective lens (NA = 0.75). A heating stage with a thermocouple attached to a GCS, functionalized with FNDs, was used to maintain SCO material **1** in the high spin-state (328 K).

### PL Spectrometer

PL spectra was acquired on a QEPRO-XR UV-NIR (250–950 nm) extended range spectrometer using a back-thinned TE cooled 1024 × 58 element charged couple detector (CCD) array using a 10 μm slit with ∼1.6 nm fwhm optical resolution. PL measurements were recorded using 550 nm LED excitation light (used in NV^–^ sensing measurements) typically from 100 × 100 μm^2^ areas.

### Transmission Electron Microscopy

BF-TEM images were acquired at 200 kV accelerating voltage on JEOL 2100+ with a Gatan Model 1095 OneView CMOS camera, and JEOL 2100F FEG TEM with a Gatan Model 1027 K3-IS direct detection camera (point resolution limit 0.25 and 0.23 nm respectively). Selected area electron diffraction measurements were taken with the JEOL 2100+ TEM and OneView Camera at 200 kV. Typically, for low e- beam flux imaging, a flux of 10^2^–10^3^ e^–^ nm^–2^ s^–1^ with a 1 s exposure time was employed. *In Situ* capture transformation of material **1:** Flux ∼5 × 10^3^ e^–^ nm^–2^ s^–1^ (total fluence after 81 s exposure ∼4 × 10^5^ e^–^ nm^–2^) at 2 fps playback speed. Image analysis was performed on the ImageJ^54^ and Gatan Digital Micrograph software.

### TEM Simulation

TEM image simulations were carried out using QSTEM, a multislice program which uses the Dirac–Fock scattering potential of Rez et al.^55,56^ A fixed number of 20 slices per molecular structure was chosen, and images were calculated with a sampling set to match experimental conditions. The defocus and aberration parameters were set according to the values used in experimental imaging. The effect of limiting electron flux to the images was conducted using a custom-made Monte Carlo program that applies noise by utilizing the Poisson statistics of electrons.

### Electron Paramagnetic Resonance Spectroscopy

EPR spectra were recorded on a Bruker EMX spectrometer using Quartz glass tubes at room temperature in the X-band. ∼ 2 mg of sample was used for each measurement.

### Infrared Spectroscopy

Attenuated total reflectance spectra was taken using a Bruker ALPHA FTIR instrument. Samples were analyzed purely in the solid state.

### Energy-Dispersive X-ray Spectroscopy

EDX spectra were acquired for samples mounted on lacey carbon Copper TEM finder grids (supplied by Agar Scientific) using an Oxford Instruments X-Max 100TLE, AZTEC software was used for data analysis.

### X-ray Photoelectron Spectroscopy

XPS was performed using a Kratos AXIS SUPRA PLUS instrument with a monochromatic Al Kα X-ray source (*h*ν = 1486.6 eV) operated at room temperature with 10 Ma emission current and 12 kV anode potential. The electron collection spot size was ca. 700 × 300 μm^2^. A pass energy of 160 eV was used for the survey scans and 20 eV for the high-resolution scans. Spectra were converted into VAMAS format for further analysis. Ar etching was performed using a Kratos Minibeam 6 operated in Ar_500_+ cluster mode at 20 keV, etch time 60 s, raster size 1 mm^2^.

### Powder X-ray Diffraction

PXRD measurements were performed using a PANalytical X’Pert Prodiffractometer equipped with a Cu–Kα radiation Source (λ = 1.5432 Å, 40 kV, 40 mA) in Bragg–Brentano geometry using a Si zero background holder. All samples were wetted with acetone to aid sample adhesion.

### Thermogravimetric Analysis

A TA Q500 Thermogravimetric Analyzer was used for the thermogravimetric analysis. All samples were analyzed using a platinum pan in the presence of air. Experimental parameters were as follows: 10 min isothermal hold at room temperature, ramp from room temperature to 1000 °C at 10 °C/min, followed by a final 10 min isothermal hold at 1000 °C. Piping was used to exhaust gas safely into a fumehood.

### Magnetic Measurements

Magnetization measurements were carried out using a Quantum Design Magnetic Property Measurement System (MPMS XL) superconducting quantum interference device (SQUID) magnetometer over a temperature range from 2 to 380 K in a 1 T dc field under field-cooled (FC) and zero-field cooled (ZFC) conditions. Diamagnetic corrections were made using Pascal’s constants. Magnetization vs field measurements were performed at 2 and 300 K, using a field range of −5 to 5 T.

### Raman Spectroscopy

Micro-Raman spectroscopy was performed using a HORIBA LabRAM HR Raman microscope. Spectra were acquired using a 785 nm laser (at ∼0.2 mW (1%) power), a 50× objective, and a 300-μm confocal pinhole. To simultaneously scan a range of Raman shifts, 300 lines mm^–1^ rotatable diffraction gratings along a path length of 800 mm were employed. Spectra were detected using a Synapse CCD detector (1024 pixels) thermoelectrically cooled to −60 °C. Before spectra collection, the instrument was calibrated using the zero-order line and a standard Si (100) reference band at 520.7 cm^–1^. The spectral resolution is better than 1.7 cm^–1^ in this configuration. Variable-temperature measurements were performed within a Linkam THMS600 stage. The sample was prepared for analysis by gently pressing a small quantity of powdered samples between two glass microscope coverslips. Once the sample, mounted on the lower coverslip, had been inserted into the Linkam stage, the stage was purged for 5 min with nitrogen, then cooled to −43 °C at a rate of 5 °C/min and held for 5 min at this temperature to equilibrate. The temperature was increased at a rate of 5 °C/min in 5 °C increments from −43 °C to +107 °C, held isothermally for 5 min, then decreased at a rate of 5 °C/min in 5 °C increments from +107 °C to −43 °C. At each temperature, the focal plane was defined using spectral-based AutoFocusing and then a spectrum over the range 65–2500 cm^–1^ acquired (110 s acquisition, 1 accumulation, 2 spectral windows). The laser beam was rastered using DuoScan to describe an ∼25 μm × 25 μm × 25 μm volume. The spectra were manually despiked within Labspec 6.5 software.

### Correlative Light-Electron Microscopy Using TEM Finder Grids

The methodology used here is reported in detail in a previous publication.^22^ The schematic experimental inverted fluorescence microscope setup is shown in Figure 1A.

### Light Illumination of Compound **2**

For bulk SQUID, EPR, PXRD, and IR measurements, a PR160–456 nm Kessil LED (400–510, 0.5 W cm^–2^) was used to illuminate crystalline powder samples for 3 h (crystals were ground prior to illumination using a pestle and mortar). Irradiance values were measured at 4 cm away from the LED using a StellarNet Black-Comet SR spectroradiometer. For single-particle NV^–^ sensing and PL spectroscopic measurements, 405 (385–425) nm light from the microscope objective (5 mW cm^–2^) for varying time (detailed in the text) was used.

## Supporting Information Available

TEM, PXRD, SQUID magnetometry, XPS, Raman spectroscopy, TGA, ODMR, MM, SAED, CLEM sensing and imaging, EDX, EELS, FTIR, and PL measurements (PDF)
In-situ TEM imaging of MOF compound **1** e^–^ beam transformations, showing Ag^0^ metal nanoparticle growth (MP4)
