# Supplementary material for: Sensing the Spin State of Room-Temperature Switchable Cyanometallate Frameworks with Nitrogen-Vacancy Centers in Nanodiamonds
Source: ACS Nano. 2024 Feb 21;18(9):7148–60. doi: 10.1021/acsnano.3c11820 (PMC10919078; doi:10.1021/acsnano.3c11820)
Supplement: Supplementary file 1 — nn3c11820_si_001.pdf [file nn3c11820_si_001.pdf]

Supplementary Information

Sensing the spin-state of room-temperature switchable cyanometallate frameworks with nitrogen-vacancy centers in nanodiamonds

Bradley T. Flinn,[a] Graham A. Rance,[b] William J. Cull,[a] Ian Cardillo-Zallo,[a] Jem Pitcairn,[a,c] Matthew J. Cliffe,[a] Michael W. Fay,[b] Ashley J. Tyler,[d] Benjamin L. Weare,[b] Craig T. Stoppiello,[e] E. Stephen Davies,[a] Melissa L. Mather*,[d] Andrei N. Khlobystov*.[a, b]

a: School of Chemistry, University of Nottingham, Nottingham, NG7 2RD, UK.

b: Nanoscale and microscale Research Centre, University of Nottingham, Nottingham, NG7 2RD, UK.

c: School of Chemistry, University of Birmingham, Birmingham, B15 2TT, UK.

d: Optics and Photonics Group, Faculty of Engineering, University of Nottingham, Nottingham, NG7 2RD, UK.

e: Centre for Microscopy and Microanalysis, University of Queensland, St. Lucia, 4072, Australia.

Contents

List of Figures 1

List of Figures

S1. TEM histogram size analysis for compounds 1 and 2a 3

S2. Powder X-ray diffractograms of compounds 1, 2a and 2b 4

S3. Thermal variation of χ_m for compound 1 showing hysteretic switching between LS and HS spin states 4

S4. Variable temperature Raman spectroscopy analysis of aged and fresh samples of SCO from compound 1 5

S5. SQUID and Raman spectroscopy analysis for 'aged' powders of compound 1 6

S6. Thermogravimetric analysis of compound 1 6

S7. A Photograph of a collection of single crystals of compound 1 with Ar^+ etching XPS analysis 7

S8. The effect of the 200 keV transmission electron beam on the PL and NV sensing properties of FNDs with increasing fluence 8

S9. Selected area electron diffraction series with tracked diffraction spot intensities 9

S10. Second Location for electron beam induced changes in NV^- sensing response 10

S11. Electron beam damage mechanism of compound 1 .... **11**

S12. Correlative light-electron microscopy NV^- sensing of Ag^0 nanoparticles **13**

S13. EDX and EELS spectra of compound 1 in 'pristine' and electron beam damaged states.......... **15**

S14. FT-IR spectrum of 2a analysed as a KBr pellet of both as-synthesised and illuminated powders **16**

S15. Wide scan XPS spectra for compound 1, compound 2a and compound 2b........................... **17**

S16. High Resolution Eu 3d XPS spectra of compound 2a.. **17**

S17. PL spectra of as-synthesised, illuminated and FND+as-synthesised microcrystalline powders of compound 2a **18**

S18. Material 2a NV^- sensing MM traces with varying illumination times **18**

S19. ODMR control measures, before and after illumination of FNDs and starting materials of compound 2a **19**

S20. ODMR spectra of aqueous 2a pipetted onto a glass slide functionalised with FNDs in both as-synthesised and phototransformed states **20**

S21. ODMR spectrum showing the photomagnetic NV^- sensing response for compound 2b **20**

S22. Thermal variation of the value $\chi_m T$ for compound 2b in both the as-synthesised and phototransformed states **21**

S23. Control ODMR Spectra of a FND cluster before and after electron beam exposure at fluence required to damage microcubes of compound 1 .... **21**

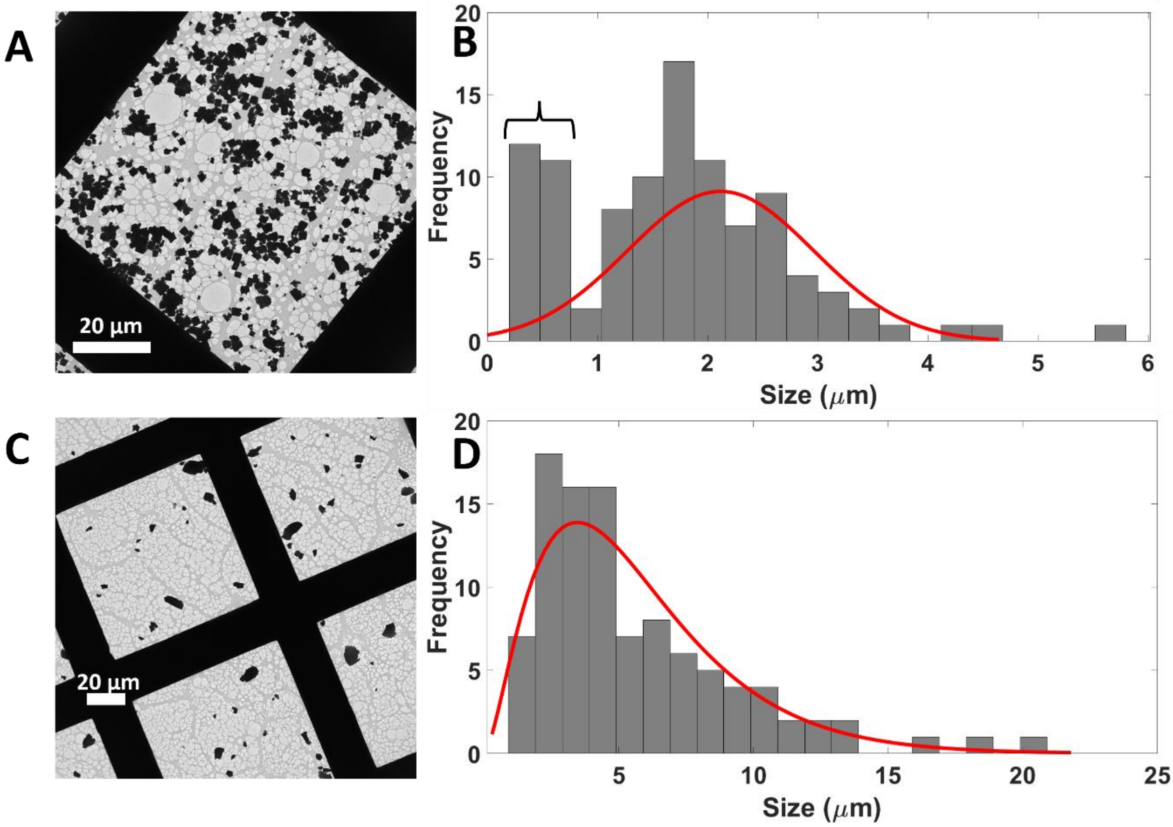

Figure S1. TEM histogram size analysis for compound **1** (B) and **2a** (D) in powdered forms. Microcrystalline **2a** and **2b** had negligible size differences. Low magnification TEM micrographs for both compound **1** (microcubes) and **2a** (microcrystals) are shown, (A) and (C) respectively. Sample size for each distribution was measured from 100 different particles taking the largest dimension of each particle. Histogram B showed a normal distribution centred at 2.1 µm. Frequency was also observed for smaller particles which were more non-uniform in morphology (data highlighted by bracket) which were not included in the normal distribution (smaller particles had the same EDX elemental composition ratios). Histogram D shows a positively skewed histogram centred at 3.5 µm.

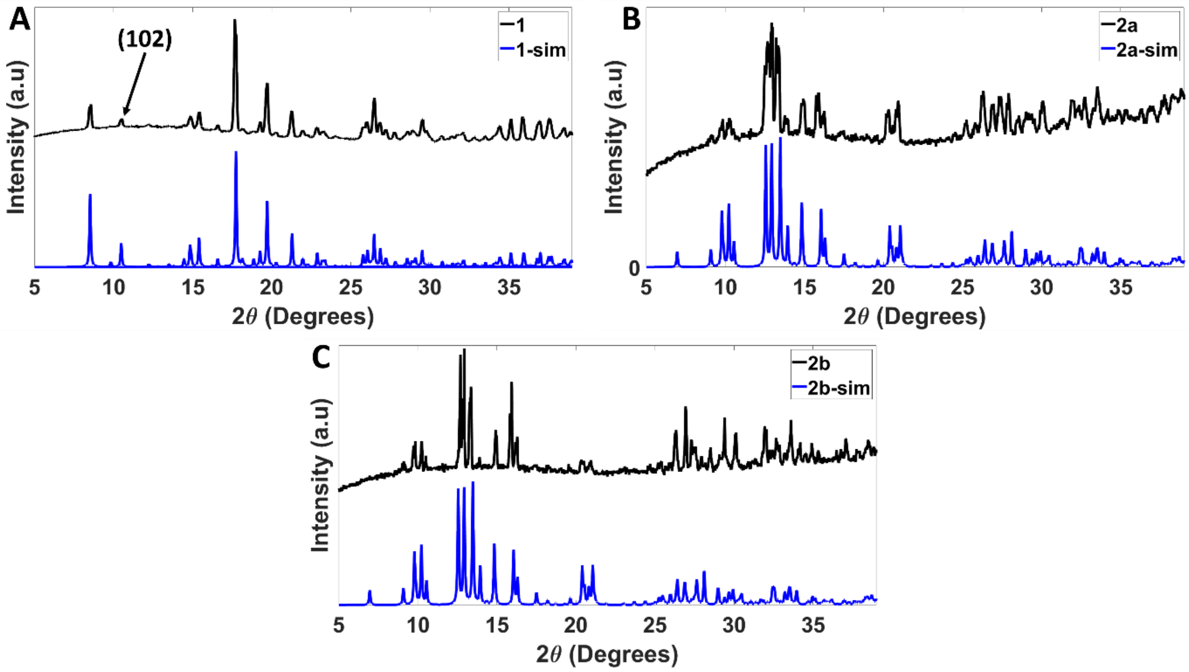

Figure S2. Experimental and simulated (sim) Powder X-ray diffractograms of compounds **1** (A), **2a** (B) and **2b** (C). Simulated diffractograms completed on Mercury software. The (102) plane for compound **1** observed in high-resolution TEM imaging is identified with an arrow.

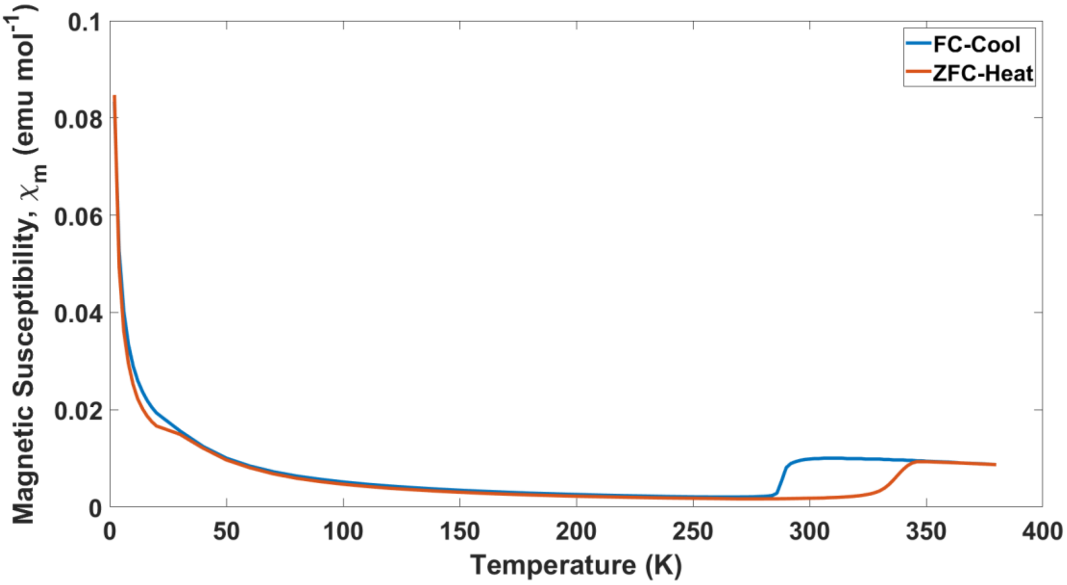

Figure S3. Thermal variation of χ_m for 'aged' compound **1** showing SCO hysteretic switching between LS and HS spin states centred approximately around room temperature (see main text for more information). Field cooled (FC) and zero field cooled (ZFC) indicate the cooling and heating cycles respectively.

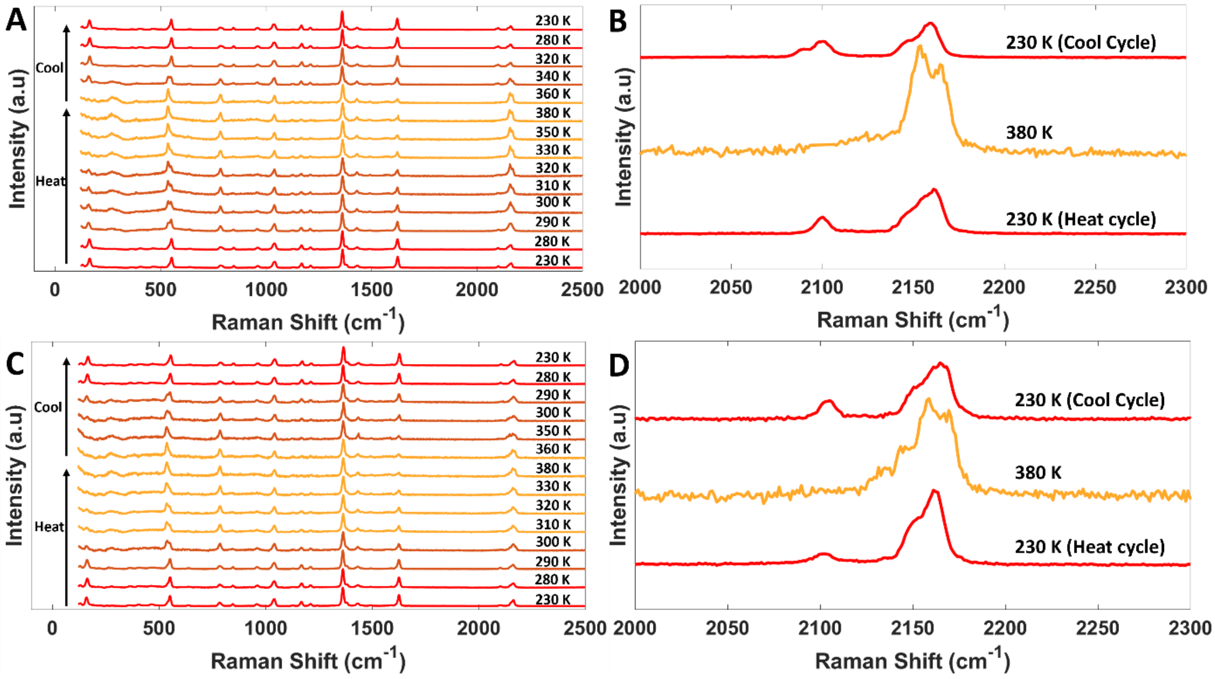

Figure S4. Variable temperature Raman spectroscopy analysis of 'aged' (A and B) and 'fresh' samples (C and D) of SCO compound **1**. Stacked spectra in A) and C) show the spectral changes with heating and cooling of 'aged' and 'fresh' **1**, respectively. Stacked spectra in B) and D) highlight the changes in the C≡N stretching modes at the start of the measurement (red, bottom), after heating above the SCO temperature to 380 K (orange, middle), and subsequent cooling below the SCO temperature (red, top), for aged and fresh **1**, respectively. Above the SCO, there is clear evidence for a change in inner sphere coordination around the Fe centre, resulting in a reduction in the C≡N peak intensity at 2098 cm^{-1} (relative to 2158 cm^{-1}). This intensity ratio (I_{2098}:I_{2158}) was used as a metric to describe the hysteretic behaviour of **1** as a function of temperature in **Figure 2**. Interestingly, 'aged' samples of **1** showed poorer reversibility in thermal behaviour, indicative of an irreversible structural change, in addition to that observed as part of SCO.

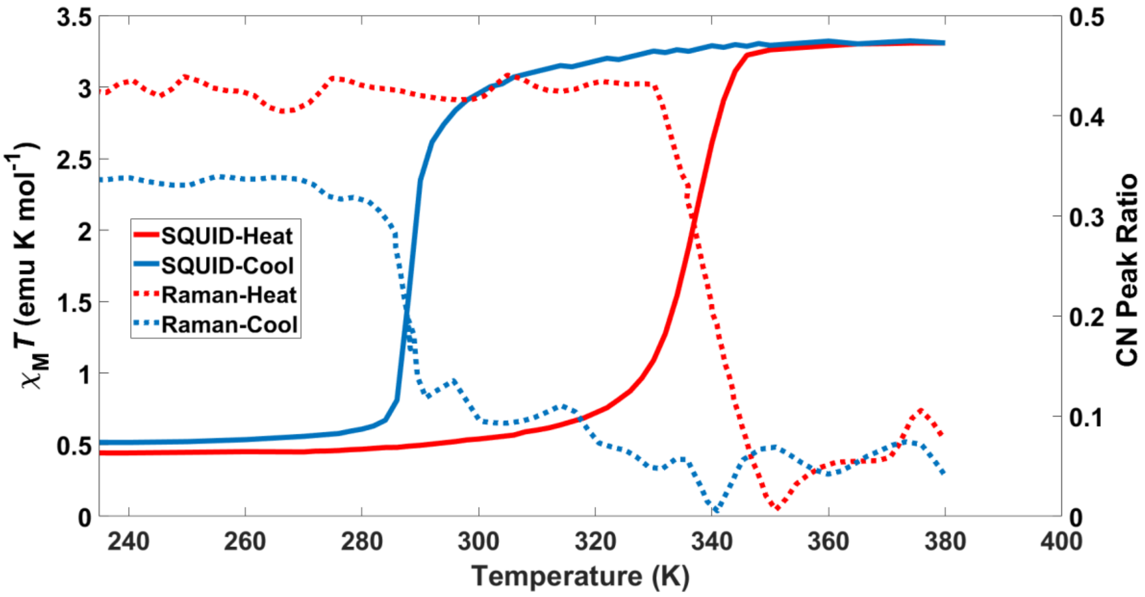

Figure S5. SQUID magnetometry, primary y-axis, and Raman spectroscopy, secondary y-axis, analysis for 'aged' powders of compound **1**. The secondary y-axis displays the intensity ratio of Raman peaks (as height) associated with C≡N stretching vibrational modes at 2098 and 2158 cm^{-1}. There is excellent correlation between the two techniques in determining the temperature of SCO (LS→HS at 338 and 338 K, SQUID and Raman, respectively; HS→LS at 288 and 287 K, SQUID and Raman, respectively).

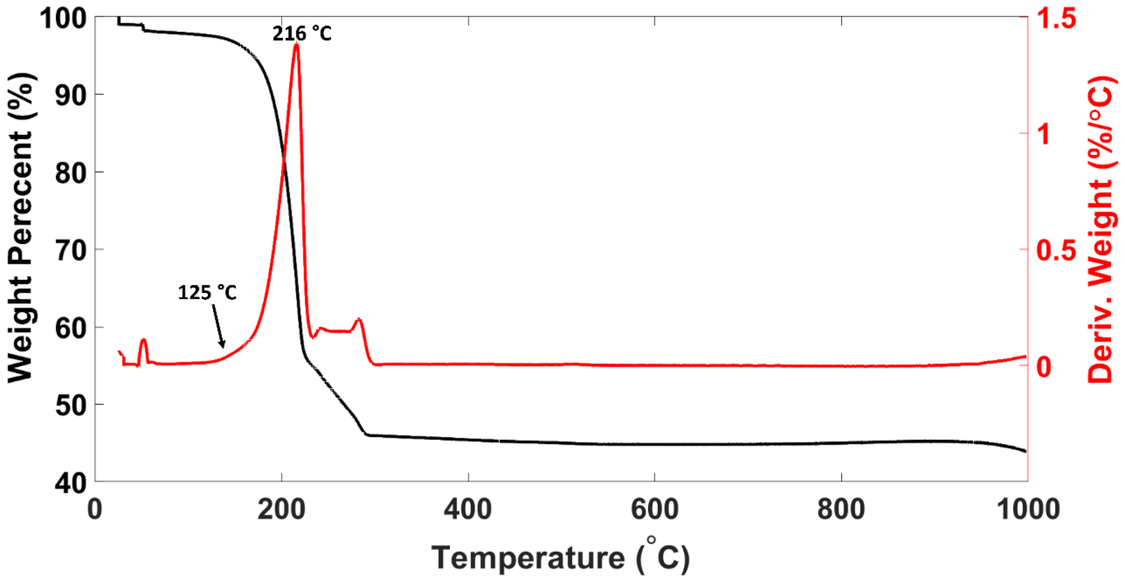

Figure S6. TGA of compound **1** showing weight percent (%) and derivative weight percent (%/°C). 10°C/min ramp rate in a platinum pan in the presence of air.

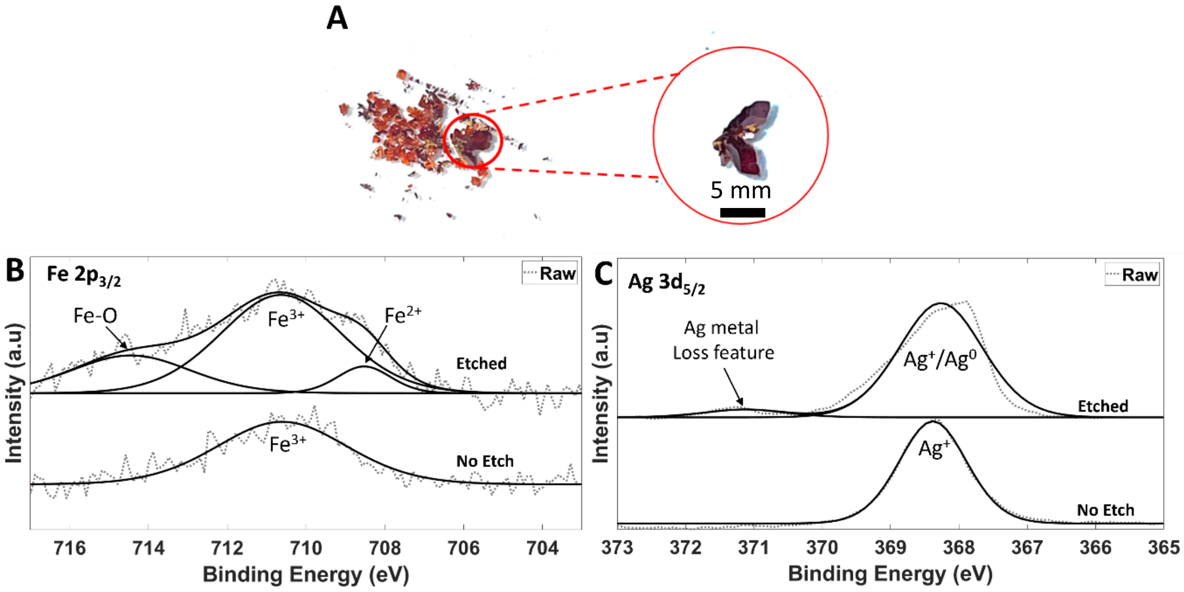

Figure S7. A) Photograph of a collection of single crystals of compound **1**. XPS analysis, Fe $2p_{3/2}$ B) and Ag $3d_{5/2}$ regions C) on the effects of Ar^{+} etching (~ 100 nm) single crystals. Etching into the crystal appears to damage the lattice structure, evidenced by formation of iron oxide (Fe-O) and silver metal (Ag^{0} metal - asymmetric $3d_{5/2}$ and loss features present).

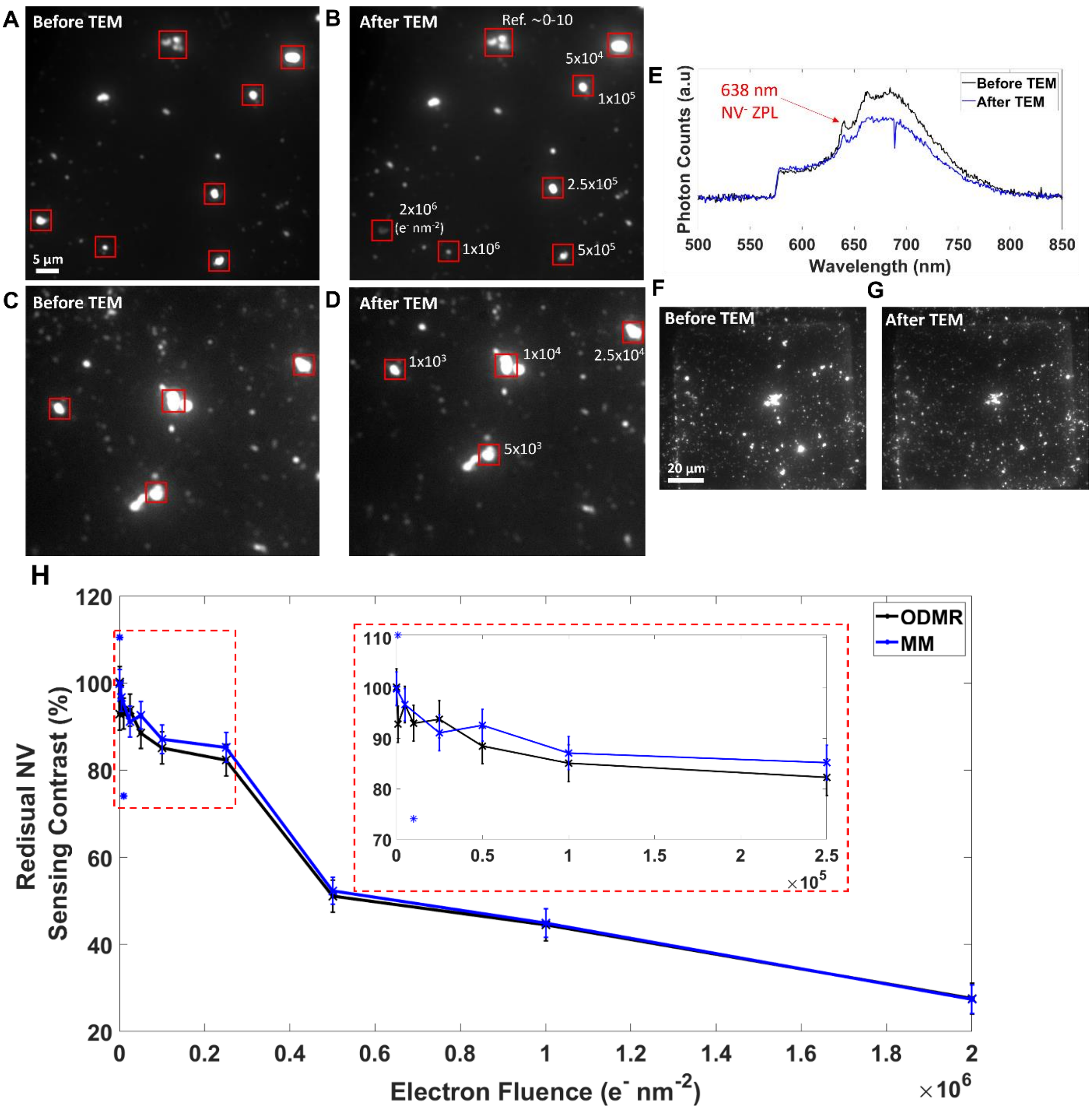

Figure S8. The effects of the 200 keV transmission electron beam on the PL and NV sensing properties of FNDs used herein. A)→B) and C)→D) PL images before and after e^- beam irradiation of several FND clusters on TEM grids at varying e^- beam fluence (stated next to clusters highlighted in red squares). Visually, especially for higher e^- beam fluence, clusters after TEM (B and D) appear less bright, indicating lower PL. E) PL spectra of a TEM grid square containing FND clusters of varying size (before and after TEM PL images is shown in F and G respectively). As expected, NV PL photon counts after e^- beam irradiation decreased. FND clusters on the grid were individually targeted and exposed to ~10^6 e^- nm^{-2}. Again, visually under the same excitation conditions, clusters in F appear brighter than G. H) A plot of residual NV^- sensing contrast vs e^- beam fluence for ODMR and MM. The red dotted square indicates low e^- fluence data points which are zoomed in the inset. Data points at 10^3 and 10^4 e^- nm^{-2} for MM showed outliers and were omitted from the general trend.

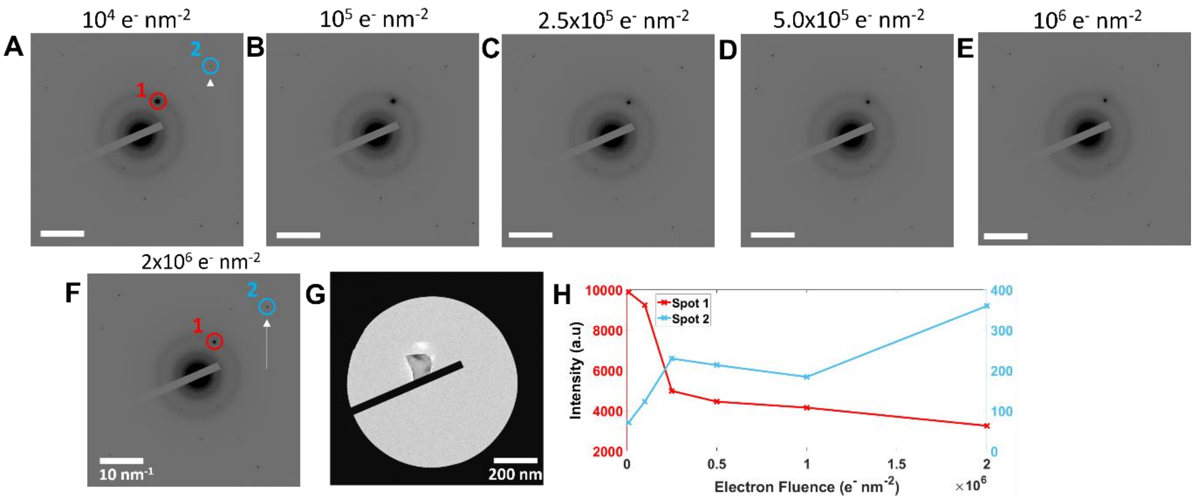

Figure S9. A-F) Diffraction series with tracked selected area electron diffraction spots (1 and 2) with increasing e^- fluence for the single FND particle show G). H) Plot of diffraction spot intensity as a function of cumulative fluence. Translation and rotation of the FND particle is manifested in the diffraction spot intensities, 1 and 2, both increasing and decreasing during irradiation. This suggests changes in crystal plane orientation with respect to the electron beam.[1]

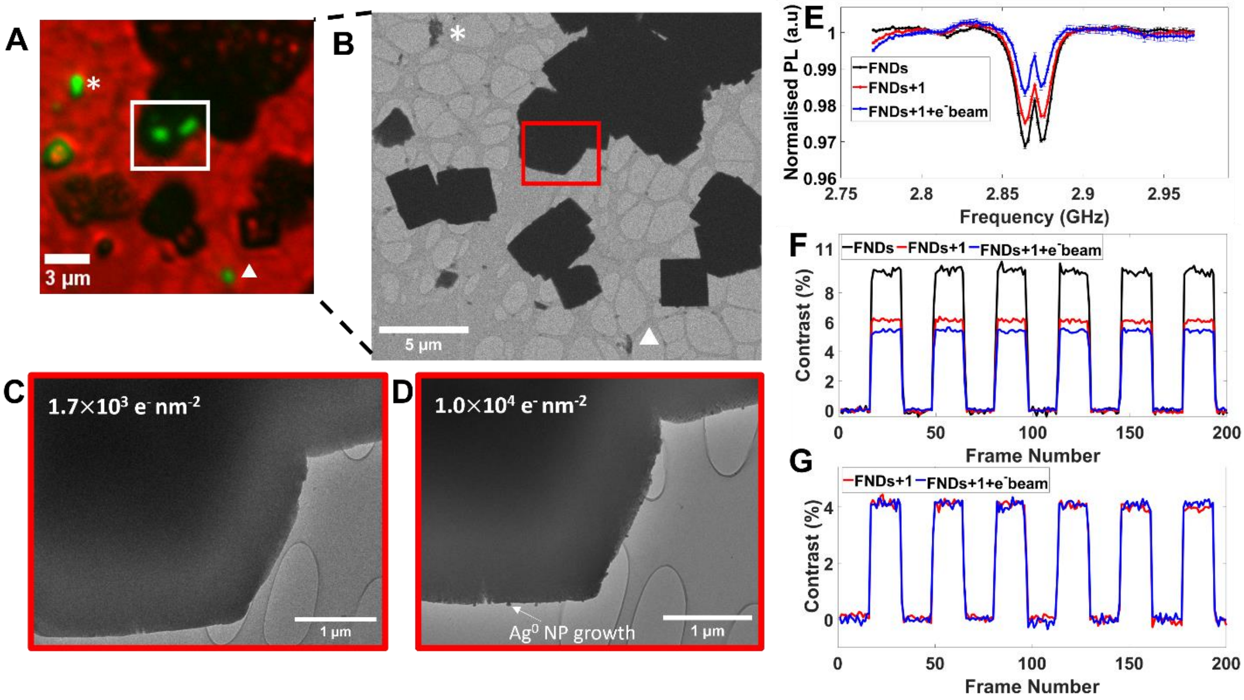

Figure S10. A) Overlayed brightfield and fluorescence images. Green spots are FND clusters, black areas correspond to deposited compound **1** as well as carbon film and red areas show the background glass coverslip. B) TEM imaging of the same area identified in optical microscopy (A). Identifying markers (white asterisk and triangle show surrounding FND clusters. As seen from the two bright green FND spots (highlighted by the white square in A, which are below microcubes of **1)**. Thickness of microcubes showed little electron transparency therefore FNDs identified optically cannot be seen via electron microscopy. C) and D) low and high e^- beam fluence images of the area marked by the red square in B), of 'pristine' and damaged states respectively. E) and F) ODMR and MM NV sensing measurements of the two FND clusters highlighted in A), before and after drop casting of **1** (black and red lines respectively) and also before and after electron beam damage of particles of **1** (red and blue lines respectively). G) A control measurement showing the MM trace of the FND cluster indicated by asterisk before and after receiving the same electron beam fluence as the target FND clusters. As seen, this is no measurable change for this FND cluster.

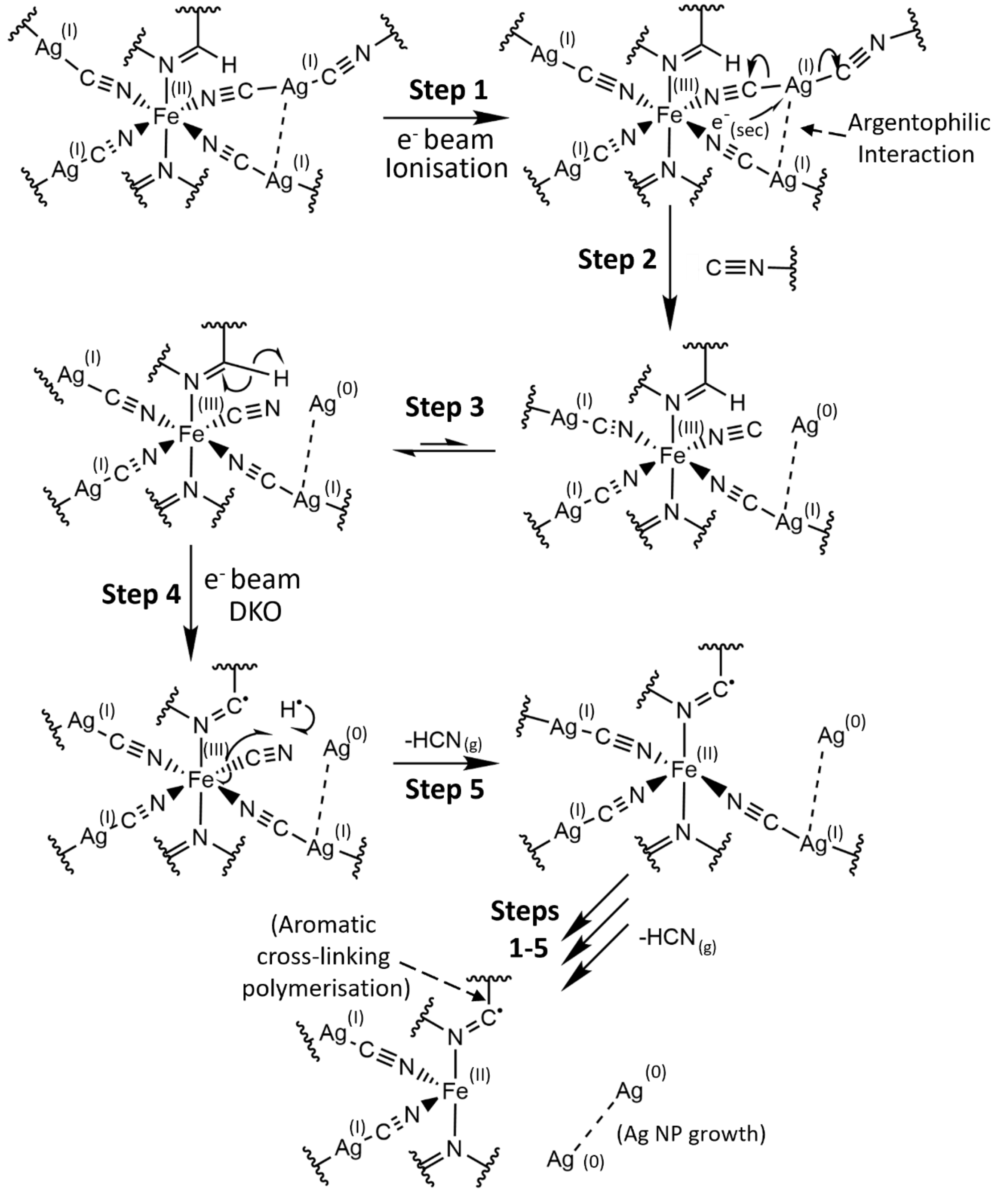

Figure S11. Proposed electron beam damage mechanism of compound **1**. Step 1 involves ionisation, Fe^{II}→Fe^{III}, of the redox active Fe centre. Secondary electron processes (radiolysis) then dominate the damage mechanism. A slower moving (relative to the direct electron beam energy, 200 keV) secondary electron, e^{-}_{sec}, then reduces silver, Ag^{I}→Ag^{0}, breaking two Ag-C coordinate bonds (step 2). Step 3 involves cyano ligand isomerisation, Fe^{III}-NC→Fe^{III}-CN. Initial Fe^{II}-NC-Ag^{I} linkage direction is determined by the precursor.[2] After silver reduction and Ag-C bond breakage, isomerisation occurs to form the more stable Fe-CN configuration. Step 4 demonstrates a ubiquitous process for hydrogen containing compounds where DKO damage cleaves C-H bonds homolytically creating hydrogen and aromatic radicals. Aromatic radicals are then likely to cross link to form an amorphous polymeric structure.[1, 3]

Step 5 demonstrates the thermodynamically favourable loss of $HCN_{(g)}$ from the terminal Fe-CN and $H^{\bullet}$ species via the curly arrow mechanism shown (loss of carbon and nitrogen during an increase of e^- fluence is evident in EDX and EELS spectra, **Figure S13**). During step 5, iron is reduced to form Fe^{II} and the steps 1-5 continue in a repeated fashion, explaining the observed Ag^0 nanoparticle growth during imaging (SI file, *in situ* TEM video).

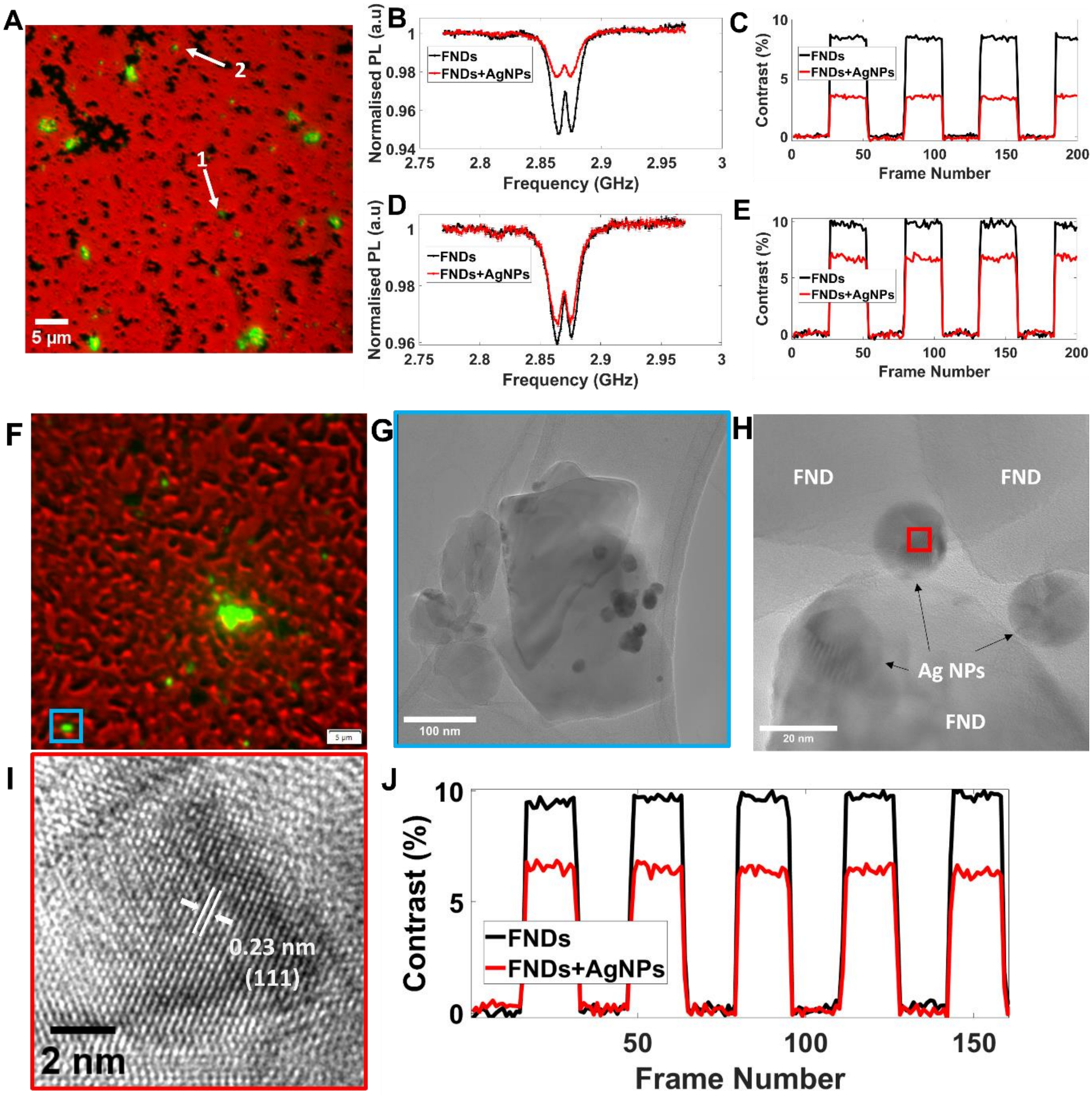

Figure S12. (A-E) and (F-J) Glass coverslip and TEM finder grid NV sensing and imaging of drop cast Ag NPs respectively. A) An overlayed optical brightfield and fluorescence image of a GCS functionalised with FNDs and drop cast Ag NPs. FNDs appear as bright green spots, black areas correspond to deposited Ag NPs (red areas are background). B) and C) show an ODMR spectrum and MM trace, respectively, of the FND cluster (location 1) highlighted by a white arrow in A). D) and E) show an ODMR spectrum and MM trace, respectively, of the FND cluster (location 2) highlighted by a white arrow in A). Visually from A) location 1 has a greater amount of Ag NPs in close proximity to the FND cluster than in location 2, this corresponds to a greater contrast reduction observed in both ODMR and MM measurements. F) An overlayed optical brightfield and fluorescence image of a location on a TEM finder grid. FNDs again appear as bright green spots, black areas correspond to carbon film and deposited Ag NPs (red areas are background). G) TEM imaging of the area marked in (F) indicated by a blue square. H) Shows typical locations on the TEM grid where Ag NPs have deposited on FND surfaces (or in very close proximity on the carbon film). I) HR-TEM imaging of the area marked by a

red square in H). d-spacing of the (111) plane is identified, marked with arrows and indexed. J) NV sensing MM trace of the FND cluster shown in the blue square in F) before and after the addition of Ag nanoparticles. As shown, there is a drop in MM contrast upon Ag NP addition, indicating the presence of spin active surface impurities on Ag NP surfaces. Correlative light-electron microscopy imaging combined with NV sensing methodology was established and previously explained.[4]

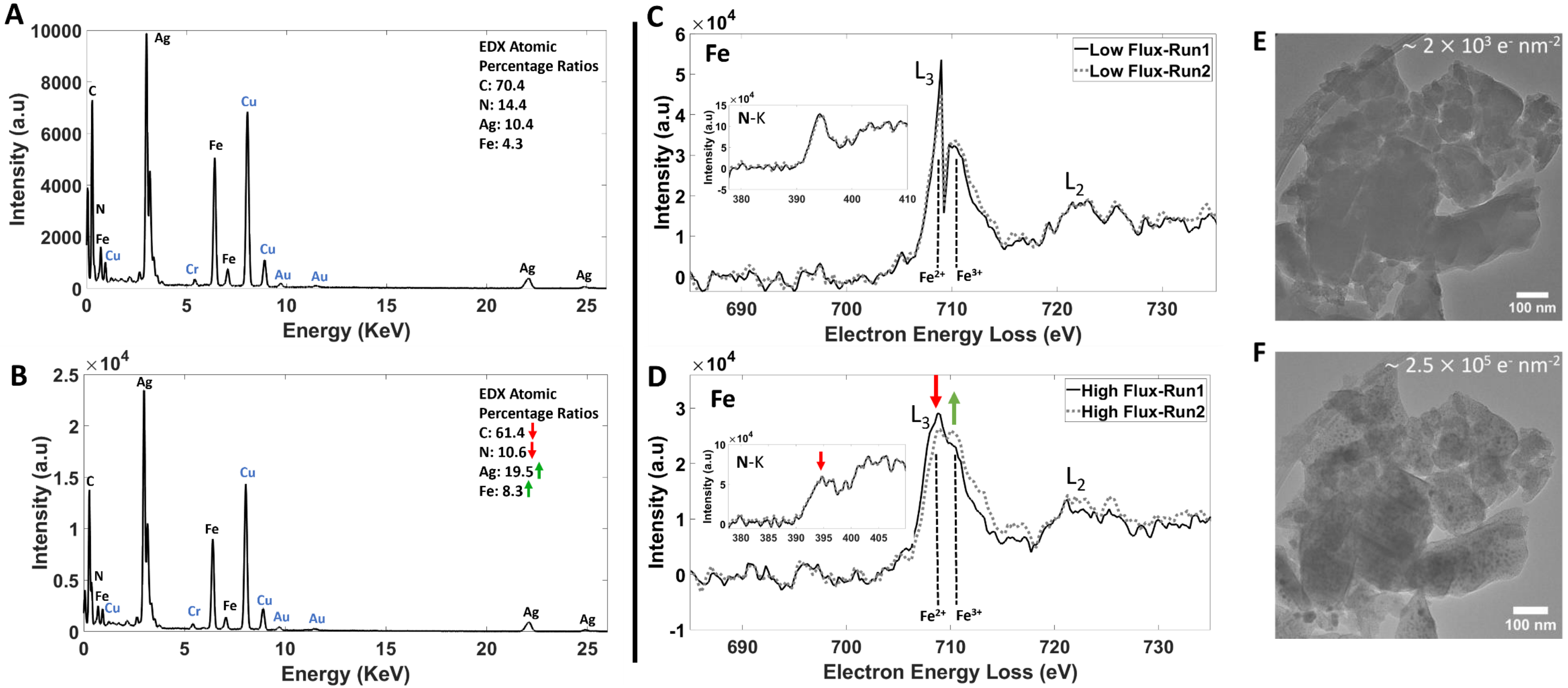

Figure S13. EDX spectra of compound **1** particles shown in Figure 6 of the main text in pristine (A) and e^- beam damaged states (B). Inset shows atomic percentage ratios of elements. Upon higher flux there is a significant decrease in carbon and nitrogen content along with an increase in metallic (Fe and Ag) content following the proposed mechanism in Figure S11. Blue labelled elements show common elemental signals found in TEM measurments (not part of compound **1**), Cu is the metallic grid frame, Au and Cr are found in the TEM holder and column. Fe EELS spectra of pristine (C) and e^- beam damaged states (D). Inset shows the N-K edge EELS spectra in both states. Upon increasing e^- fluence there is a significant change in the Fe L_3 loss peaks corresponding to Fe oxidation state change as indicated by red and green arrows. There is a also a diminshing of the N-K edge at higher fluence consistent with loss of N in the structure, proposed from loss of $HCN_{(g)}$. TEM images of the pristine (E) and e^- beam damaged area (F) taken for EELS capture respecitvely, inset shows the total fluence values.

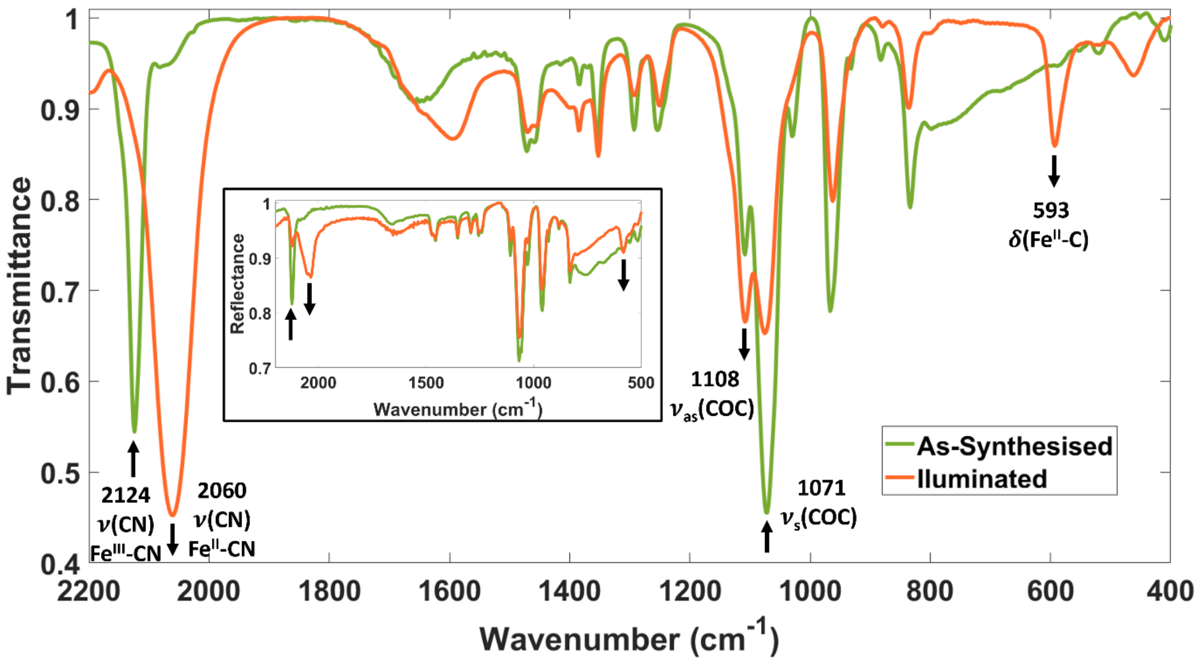

Figure S14. FT-IR spectrum of **2a** analysed as a KBr pellet of both as-synthesised and illuminated powders. (inset shows **2a** analysed by ATR-IR). Pellet and powders were illuminated for 2 hours. Incomplete phototransformation (i.e the presence of remaining $Fe^{III}CN$ in the illuminated state) was observed for ATR-IR power measurements, which was not observed for KBr measurements. For KBr measurements the crystals of **2a** were ground in a pestle and mortar (greater dispersion of smaller particles in the KBr disc). Illumination and therefore photoconversion of material will depend on the penetration depth of the light into the crystal.[5]

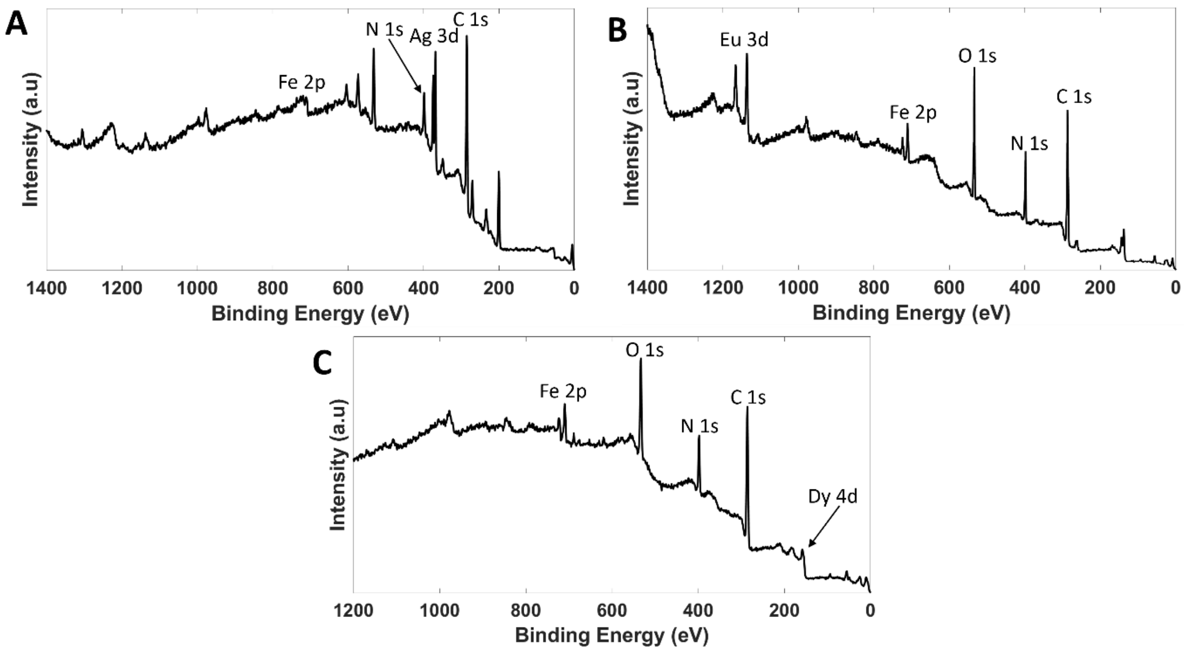

Figure S15. Wide scan XPS spectra for compound **1**, compound **2a** and compound **2b**, A, B and C respectively.

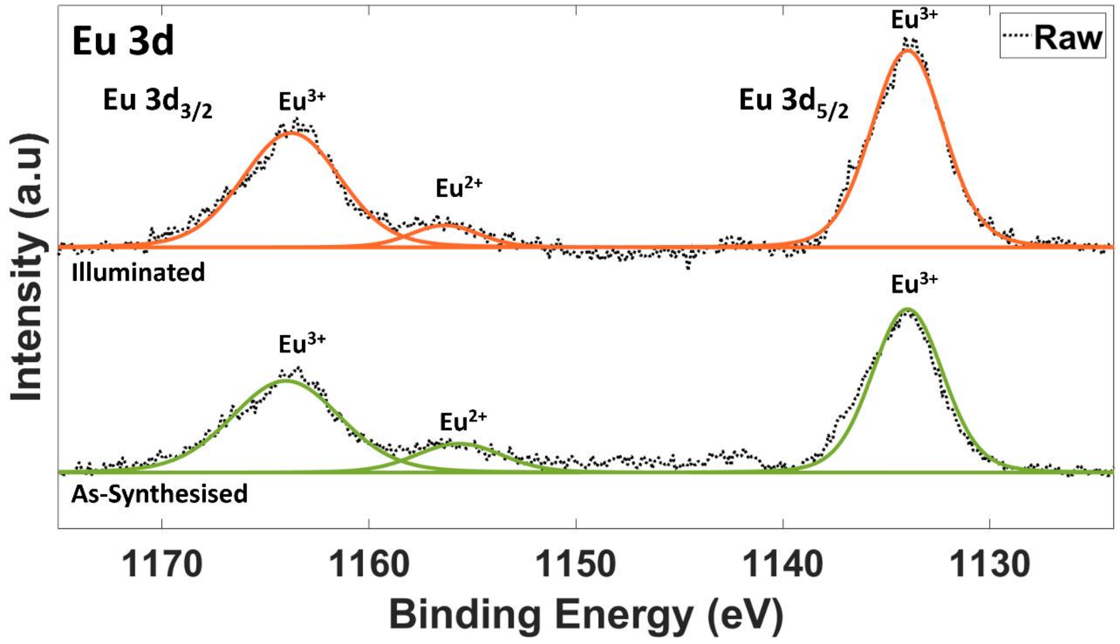

Figure S16. High Resolution Eu 3d XPS spectra of compound **2a**. Upon illumination there is no evidence for Eu oxidation sate change, consistent with the proposed photoinduced electron transfer mechanism. A small shoulder in the Eu $3d_{5/2}$ region potentially corresponding to Eu^{2+} is shown, in line with previous reports.

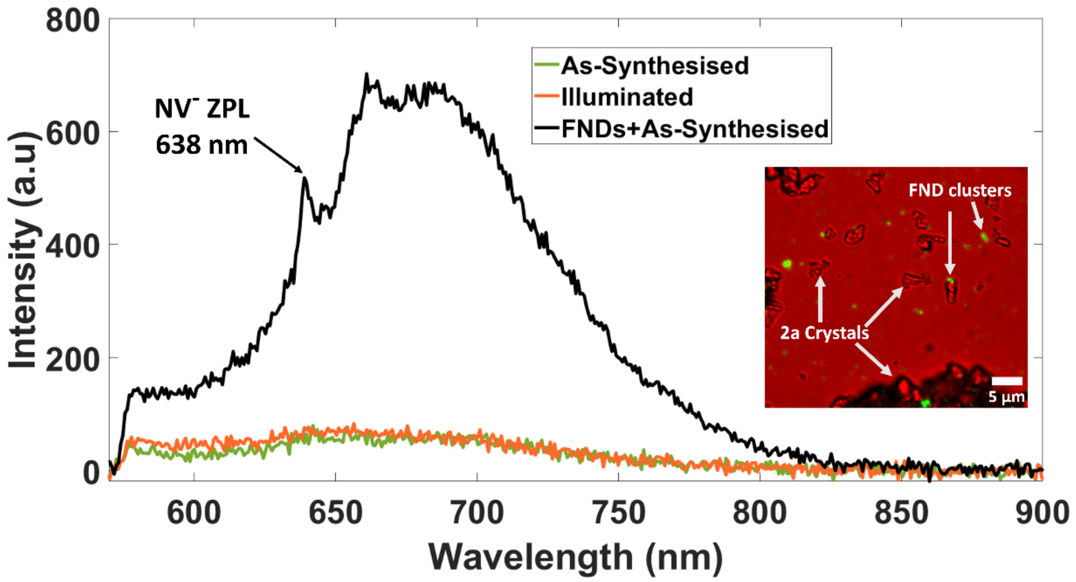

Figure S17. PL spectra recorded for as-synthesised, illuminated and FND+as-synthesised microcrystalline powders of compound **2a** of a typical area of analysis**.** The NV^- zero phonon line (ZPL) is indicated (638 nm). PL was collected using 550 nm excitation light from a 100 x 100 μm^2 area. Inset optical image is an overlayed brightfield and fluorescence image of typical areas of analysis where there is FND clusters/single particles (green bright spots) and microcrystals of **2a** as indicated by white arrows.

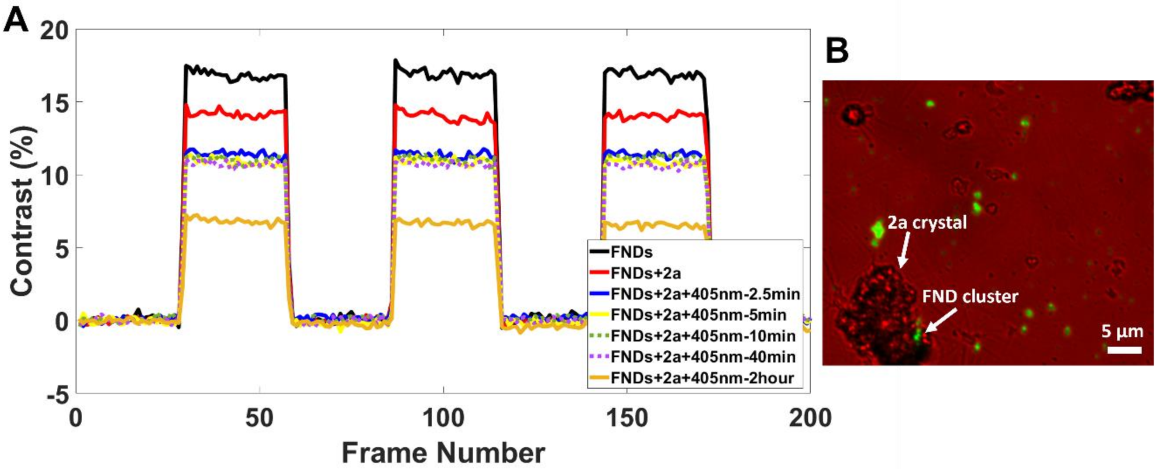

Figure S18. A) MM traces at different illumination times (405 nm from the objective, 5mW cm^{-2}) for the FND cluster highlighted by a white arrow in B. B) Overlayed brightfield and fluorescence image with arrows to show FNDs and crystals of **2a** which are in close proximity.

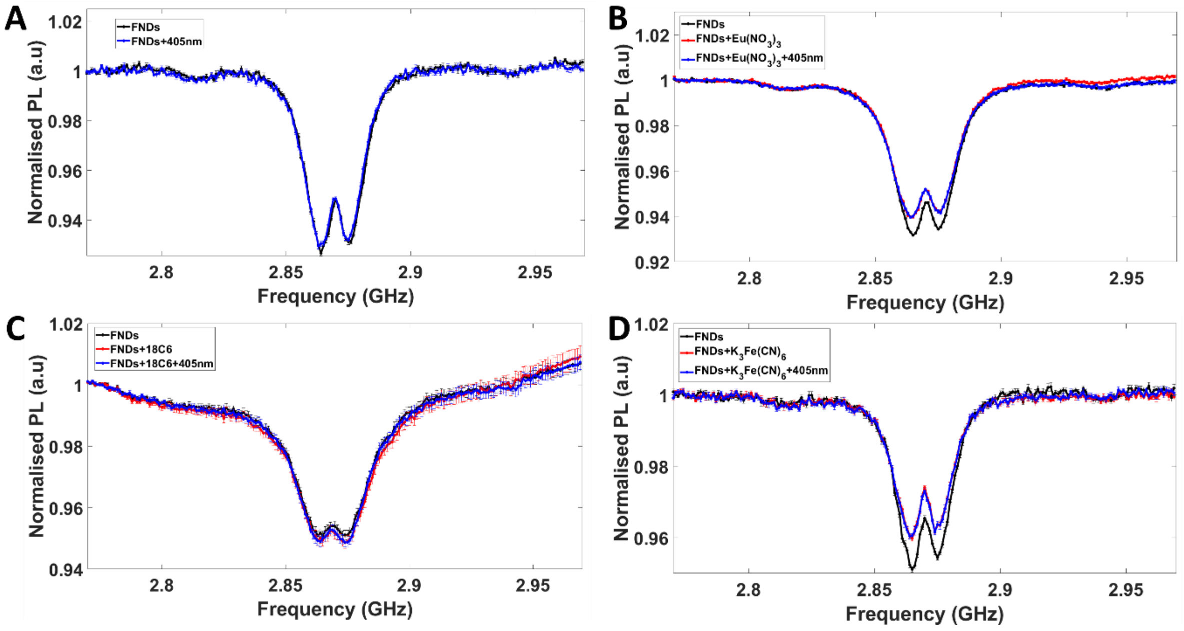

Figure S19. ODMR spectra control measures, before and after illumination of FNDs and precursors of compound **2a**. A = FNDs, B = $Eu(NO_3)_3$, C =18C6 (18-crown-6) and D = $K_3Fe(CN)_6$. All materials show no response upon illumination. All chemical precursors were drop cast with ethanol and dried. Addition of $Eu(NO_3)_3$ and $K_3Fe(CN)_6$ to FNDs both yielded ODMR contrast reduction due to paramagnetic Eu^{III} and Fe^{III}-(LS) metal centres respectively. 18C6 showed no observable ODMR contrast reduction upon addition due to being diamagnetic.

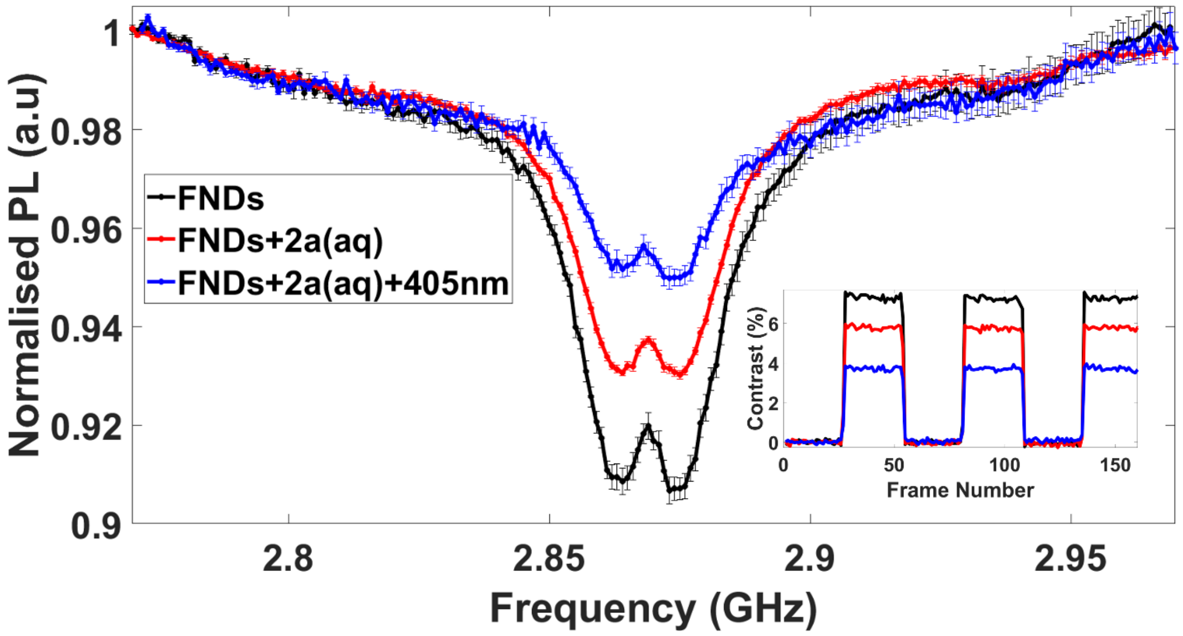

Figure S20. ODMR spectra of aqueous **2a** (1 mg mL^{-1}) pipetted (200 µL) onto a glass coverslip functionalised with FNDs (black trace shows background signal of FNDs+Water). Inset shows MM traces. Illumination (10 minutes using 405 nm light from the objective, see experimental) shows the same trends as solid-state sensing measurements, confirming the PET mechanism between aqueous cationic $[Eu(18C6)(H_2O)_3]^{3+}$ and anionic $[Fe(CN)_6]^{3-}$ anions persists.

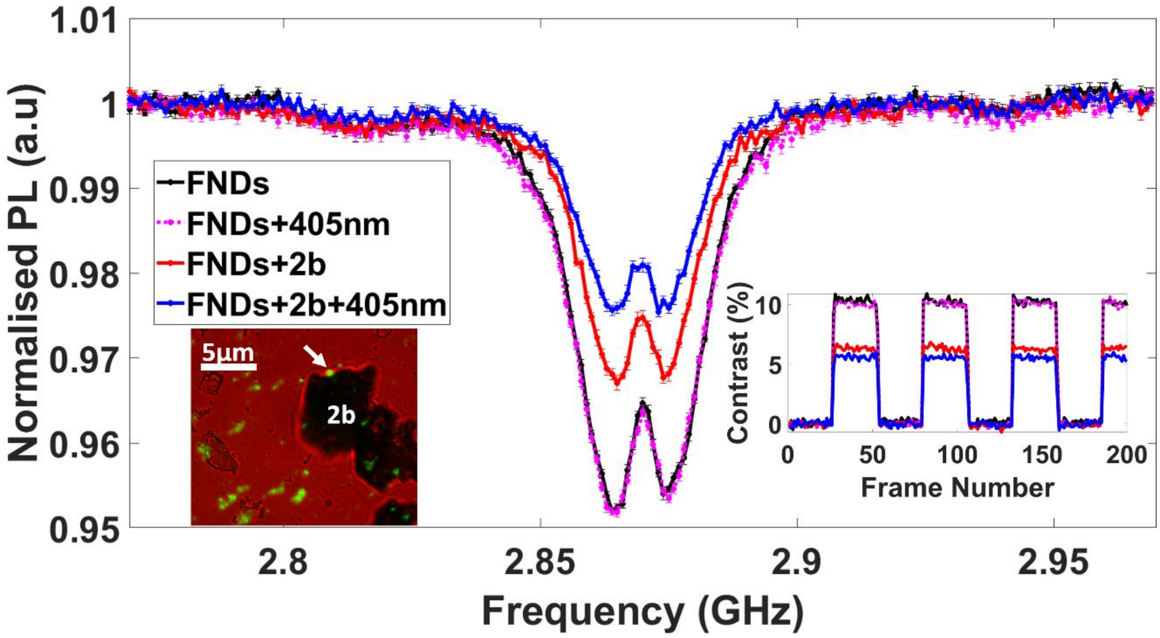

Figure S21. ODMR spectra showing the solid-state photomagnetic NV^- sensing response for compound **2b**. The control of FNDs+405 nm without **2b** shows no response under the light conditions (magenta dotted line). Inset left: Overlayed optical brightfield and fluorescence image showing the FND cluster analysed which is contact/close proximity to a crystal of 2b on a glass coverslip. Inset right: MM traces showing the solid-state photomagnetic NV^- sensing response.

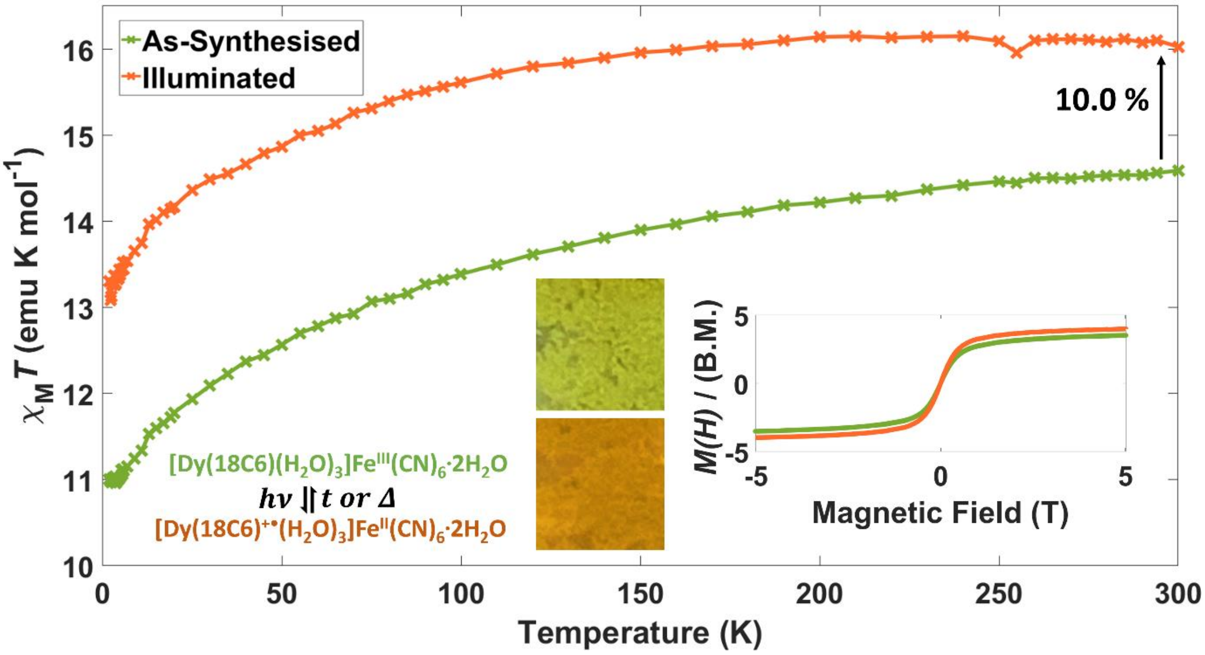

Figure S22. Thermal variation of the value $\chi_m T$ for compound **2b** in both the as-synthesised (green) and illuminated (orange) states. Inset photographs show powders of **2b** in both states. Inset equation also shows the chemical formula of the reversible states. Light, $h\nu$ is used to phototransform the powder and time (> 1 week), t or heat (Δ, 80 °C for 2 hours), can be used to reverse the phototransformation. Inset *M(H)* curve for the as-synthesised (green) and illuminated (orange) powders at 2 K is also shown.

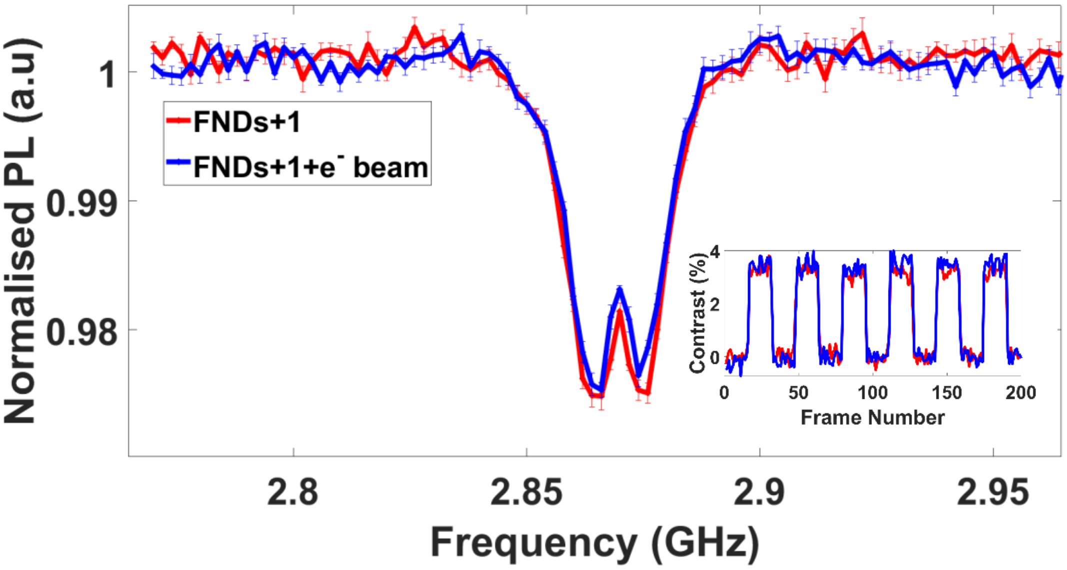

Figure S23. Control ODMR spectra and MM trace of a FND cluster before and after electron beam irradiation, which is not in close proximity to microcubes of **1**. The e^- beam fluence used is enough to damage microcubes of compound **1** (~ 1×10^4 e nm^{-2}). At this electron fluence, e^- beam induced NV PL changes in FND particles is negligible/within experimental error (see **Figure S8**).

References

(1) Fung, K. L. Y.; Weare, B. L.; Fay, M. W.; Argent, S. P.; Khlobystov, A. N. Reactions of polyaromatic molecules in crystals under electron beam of the transmission electron microscope. *Micron* **2023**, *165*.

(2) Shatruk, M.; Dragulescu-Andrasi, A.; Chambers, K. E.; Stoian, S. A.; Bominaar, E. L.; Achim, C.; Dunbar, K. R. Properties of prussian blue materials manifested in molecular complexes: Observation of cyanide linkage isomerism and spin-crossover behavior in pentanuclear cyanide clusters. *Journal of the American Chemical Society* **2007**, *129*, 6104-6116.

(3) Chamberlain, T. W.; Biskupek, J.; Skowron, S. T.; Markevich, A. V.; Kurasch, S.; Reimer, O.; Walker, K. E.; Rance, G. A.; Feng, X. L.; Mullen, K.; et al. Stop-Frame Filming and Discovery of Reactions at the Single-Molecule Level by Transmission Electron Microscopy. *Acs Nano* **2017**, *11*, 2509-2520.

(4) Flinn, B. T.; Radu, V.; Fay, M. W.; Tyler, A. J.; Pitcairn, J.; Cliffe, M. J.; Weare, B. L.; Stoppiello, C. T.; Mather, M. L.; Khlobystov, A. N. Nitrogen vacancy defects in single-particle nanodiamonds sense paramagnetic transition metal spin noise from nanoparticles on a transmission electron microscopy grid. *Nanoscale Advances* **2023**.

(5) Abouelwafa, A. S.; Mereacre, V.; Balaban, T. S.; Anson, C. E.; Powell, A. K. Photo- and thermally-enhanced charge separation in supramolecular viologen-hexacyanoferrate complexes. *Crystengcomm* **2010**, *12*, 94-99.
